# ALOX5 deficiency contributes to bladder cancer progression by mediating ferroptosis escape

**DOI:** 10.1038/s41419-023-06333-7

**Published:** 2023-12-07

**Authors:** Tianyao Liu, Xinyan Xu, Jiazheng Li, Ming Bai, Wenjie Zhu, Yanqing Liu, Siyang Liu, Zihan Zhao, Tianhang Li, Ning Jiang, Yuhao Bai, Qingyang Jin, Yulin Zhang, Yufeng Zheng, Shengkai Zhou, Shoubin Zhan, Ying Sun, Gaoli Liang, Yang Luo, Xi Chen, Hongqian Guo, Rong Yang

**Affiliations:** 1 Nanjing Drum Tower Hospital, Affiliated Hospital of Medical School, Nanjing University, Nanjing, China; 2 Department of Urology, Nanjing Drum Tower Hospital Clinical College of Nanjing University of Chinese Medicine, Nanjing, China; 3 Jiangsu Engineering Research Center for microRNA Biology and Biotechnology, State Key Laboratory of Pharmaceutical Biotechnology, School of Life Sciences, Nanjing University, Nanjing, China; 4 Department of Urology, Nanjing Drum Tower Hospital Clinical College of Jiangsu University, Nanjing, China

**Keywords:** Tumour-suppressor proteins, Cell death

## Abstract

Ferroptosis is an iron-dependent form of regulated cell death driven by the lethal lipid peroxides. Previous studies have demonstrated that inducing ferroptosis holds great potential in cancer therapy, especially for patients with traditional therapy failure. However, cancer cells can acquire ferroptosis evasion during progression. To date, the therapeutic potential of inducing ferroptosis in bladder cancer (BCa) remains unclear, and whether a ferroptosis escape mechanism exists in BCa needs further investigation. This study verified that low pathological stage BCa cells were highly sensitive to RSL3-induced ferroptosis, whereas high pathological stage BCa cells exhibited obviously ferroptosis resistance. RNA-seq, RNAi-mediated loss-of-function, and CRISPR/Cas9 experiments demonstrated that ALOX5 deficiency was the crucial factor of BCa resistance to ferroptosis in vitro and in vivo. Mechanistically, we found that ALOX5 deficiency was regulated by EGR1 at the transcriptional level. Clinically, ALOX5 expression was decreased in BCa tissues, and its low expression was associated with poor survival. Collectively, this study uncovers a novel mechanism for BCa ferroptosis escape and proposes that ALOX5 may be a valuable therapeutic target and prognostic biomarker in BCa treatment.

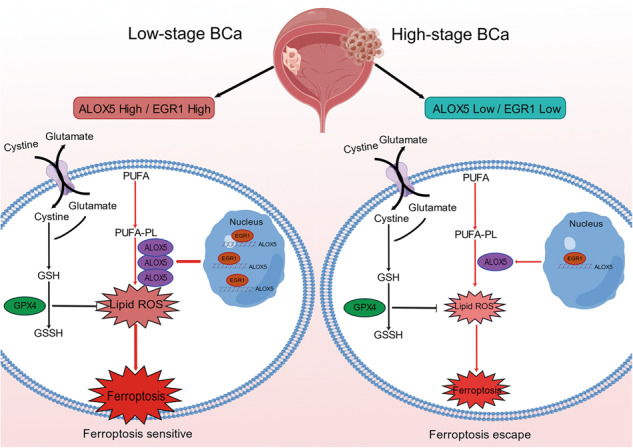

## Introduction

Bladder cancer (BCa) is one of the common malignant tumors of the urinary system, posing a serious global health threat. In 2020, it was estimated that there were 573,278 new cases and 212,536 deaths worldwide [1]. Although 70–80% of patients are initially diagnosed with non-muscle invasive BCa (NMIBC), within 5 years, 31–78% will experience a relapse and 10–15% will progress to muscle-invasive BCa (MIBC), resulting in a reduced 5-year survival rate of 36–48% [2]. Currently, cisplatin-based combination chemotherapy remains the first-line treatment for MIBC. However, only 40% patients benefit from it due to chemotherapy resistance and serious side-effects [3]. Recently, a series of clinical trials have shown that immunotherapy has achieved excellent results in prolonging overall survival rate of patients with advanced BCa [4–6]. Nevertheless, the efficacy of immunotherapy is largely limited by the complex tumor heterogeneity and immune-related adverse effects, resulting in an objective response rate to PD-1/PD-L1 monotherapy of less than 30% [7, 8]. Therefore, in-depth exploration and understanding of the molecular mechanism of BCa carcinogenesis and progression are essential to develop new therapeutic strategies to prolong the survival time of patients with advanced cancer.

Ferroptosis, an iron-dependent form of regulated cell death driven by the accumulation of lethal lipid peroxides, was discovered and named by the Stockwell laboratory in 2012 [9–12]. There are three main mechanisms that trigger ferroptosis: iron metabolism imbalance, lipid metabolism dysfunction, and antioxidant system disorder [13–18]. Accumulating evidences have shown that ferroptosis was implicated in multiple disorders, including neurodegenerative diseases, ischemia-reperfusion injury, and cancers [19, 20]. In cancers, ferroptosis plays a pivotal role in the entire process of tumorigenesis, growth, and metastasis [21–24]. Theoretically, cancer cells are more vulnerable to ferroptosis due to their distinctive metabolic characteristics, including a high load of reactive oxygen species (ROS) and an excess of labile iron [9, 25]. Thus, inducing ferroptosis holds great potential in cancer therapy, especially for patients with chemotherapy resistance and targeted therapy failure [26–29]. In addition, ferroptosis can also synergistically suppress tumor growth with traditional chemotherapy, radiotherapy, and immunotherapy [29–31]. However, cancer cells can acquire ferroptosis escape via reducing lipid ROS levels or enhancing antioxidant capacity during progression [21–23, 32], greatly attenuating the efficacy of ferroptosis-based therapy. Based on this, illustrating the mechanism of ferroptosis escape is crucial for the development of new therapeutic strategies.

Arachidonate lipoxygenases (ALOXs), are major regulators that catalyze polyunsaturated fatty acid to lipid hydroperoxides, resulting in cell membrane damage and ferroptosis. The mammalian lipoxygenase family contains six isoforms (ALOXE3, ALOX5, ALOX12, ALOX12B, ALOX15, and ALOX15B), and catalyze different lipid compounds [14, 33, 34]. Previous studies have shown that dysfunction of ALOXs is associated with resistance to ferroptosis in various cancers [35–37]. However, whether ALOXs mediate ferroptosis escape in BCa remains an open question.

Herein, we found that BCa cells with low-stage (5637 and T24) were sensitive to ferroptosis inducer (FIN) treatment, whereas those with high-stage (UMUC3 and J82) exhibited obviously ferroptosis resistance. Treatment with RSL3 significantly delayed tumor growth in low-stage BCa in vivo. Using RNA-seq and CRISPR/Cas9 technologies, we demonstrated that ALOX5 deficiency was associated with BCa progression and mediated ferroptosis escape. Mechanistically, ALOX5 deficiency may be regulated by the early growth response 1 (EGR1) at the transcriptional level. Together, our study uncovers a new mechanism for BCa ferroptosis escape and suggests that ALOX5 can serve as a prognostic indicator and potential therapeutic target.

## Results

### Ferroptosis induction suppresses proliferation, migration, and invasion of BCa cells in vitro

**Fig. 1:**
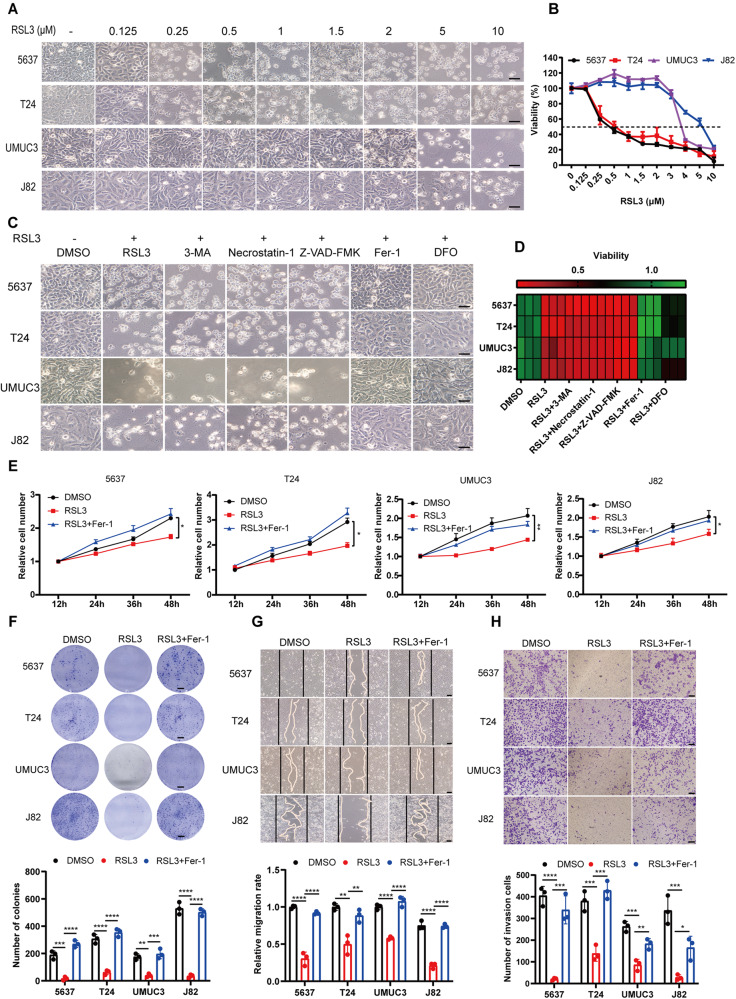
Ferroptosis induction suppresses proliferation, migration, and invasion of BCa cells in vitro. **A** Representative images of BCa cell lines (5637, T24, UMUC3, and J82) treated with various concentrations of RSL3 (0, 0.125, 0.25, 0.5, 1, 2, 5, and 10 μM) for 24 h. Scale bars, 50 μm. **B** Viability (%) of BCa cell lines treated with RSL3 at different concentrations for 24 h. **C** Ferroptosis rescue experiment of BCa cell lines treated with RSL3 in combination with various inhibitors: autophagy inhibitor (3-MA, 2 mM), necroptosis inhibitor (Necrostatin-1, 10 μg/ml), apoptosis inhibitor (Z-VAD-FMK, 10 μg/ml), ferroptosis inhibitor (Fer-1, 2 μM; DFO, 100 μM). Scare bar, 50 μm. **D** Viability (%) of BCa cell lines treated with RSL3 and rescued by various inhibitors for 24 h. **E** CCK-8 proliferation assay of BCa cells pretreated with RSL3 at suitable concentrations (5637 and T24: 0.125 μM RSL3; UMUC3: 2 μM; J82: 3 μM) that did not affect cell viability. **F** Colony formation assay. BCa cells were pretreated with RSL3 and grown for ten days. Scale bars, 5 mm. **G**, **H** The migratory and invasive ability of BCa cells were assessed by wound healing and transwell invasion assays. BCa cells were pretreated with suitable concentration of RSL3, similar to **E**). Scale bars, 50 μm. *p < 0.05, **p < 0.01, ***p < 0.001, ****p < 0.0001. The data are represented as mean ± SD of three independent assays.

Previous studies have demonstrated that cancer cells exhibit increased vulnerability to ferroptosis, and ferroptosis induction has emerged as a promising therapeutic strategy for cancers [9, 25–29]. The sensitivity of BCa cells to ferroptosis induction, however, has not been fully elucidated. To characterize the response of BCa cells to ferroptosis induction, we treated BCa cells at different pathological stages with RSL3 (a GPX4 inhibitor) at varying doses. We observed that all BCa cell lines were suffered from RSL3-induced ferroptosis (Fig. 1A, B), and could be rescued by ferroptosis inhibitor DFO and Fer-1, but not by other inhibitors such as 3-MA (autophagy inhibitor), Z-VAD-FMK (apoptosis inhibitor), or Necrostatin-1 (necroptosis inhibitor) (Fig. 1C, D). This led us to speculate whether the biological function of BCa cells were affected by ferroptosis induction prior to ferroptosis being triggered. To this end, we tested cell proliferation, migration, and invasion by pretreating cells with RSL3 at a concentration that did not affect cell viability. As expected, treatment with RSL3 significantly attenuated proliferation and colony formation in all BCa cell lines, and suppressed tumor cell migration and invasion, while this inhibitory effect could be rescued by Fer-1 (Fig. 1E–H). Taken together, these results suggest that BCa cells are responsive to ferroptosis induction, and ferroptosis may play a pivotal role in carcinogenesis and progression of BCa.

### High pathological stage BCa cells exhibit notably resistance to ferroptosis

**Fig. 2:**
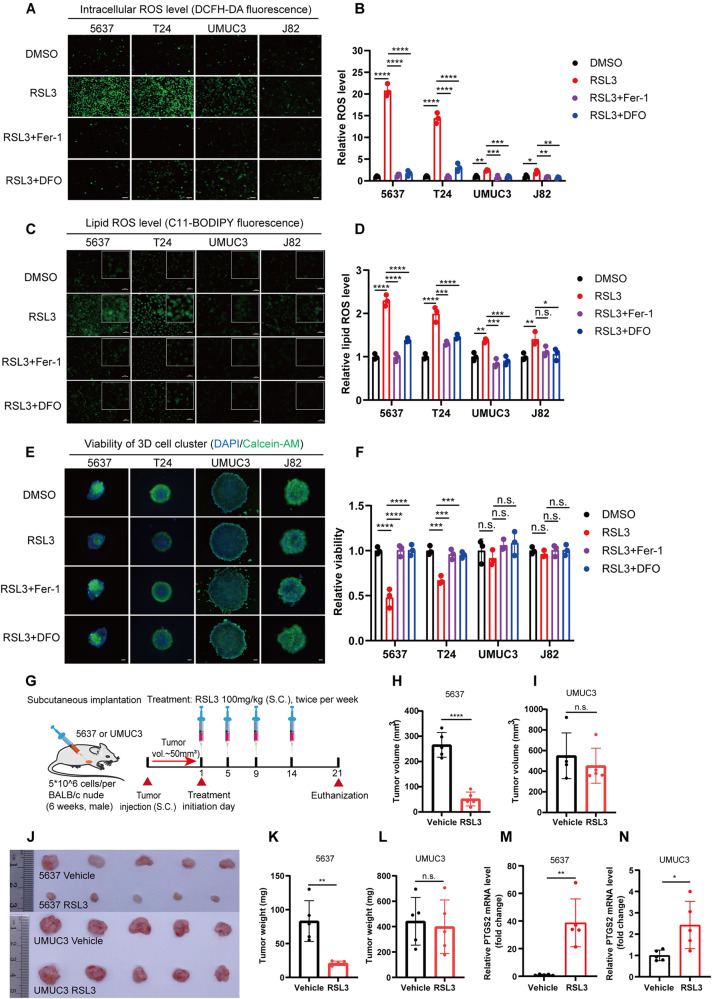
High pathological stage BCa cells exhibit notably resistance to ferroptosis. **A**–**D** The Intracellular total ROS and lipid ROS levels were evaluated using DCFH-DA and C11-BODIPY probe, respectively. BCa cells were treated with RSL3 (1 μM) and ferroptosis inhibitor for 6 h, and fluorescence was observed using a fluorescence microscope. Fluorescence intensity was measured using a fluorescence microplate reader. Scale bars, 50 μm. **E** 3D tumor spheroids were cultured for three days and then treated with RSL3 (1 μM) and ferroptosis inhibitor for 48 h. After that, the living cells was evaluated using Calcein-AM staining. Living cells were represented by green fluorescence, while DAPI staining indicated the nuclei (blue fluorescence). Scale bars, 200 μm. **F** The 3D cell viability was assessed using CCK-8 kits. **G** Schematic diagram illustrating the construction of the xenograft tumor model and the dosage regimen, *n* = 5. **H**, **I** Tumor volume measurements after 21 days of treatment with RSL3 and vehicle. **J**–**N** Representative images of xenograft tumors after treatment with RSL3 and vehicle control, along with statistical analyses of tumor weight and PTGS2 expression levels. n.s., represent no significance, *p < 0.05, **p < 0.01, ***p < 0.001, ****p < 0.0001. The data are represented as mean ± SD of three independent assays.

Ferroptosis resistance is a crucial factor that undermines the effectiveness of ferroptosis-based therapies. In BCa, we noticed that not all cell lines are vulnerable to ferroptosis at low concentrations of RSL3. Compared to low-stage BCa cells (5637 and T24), the ferroptosis resistance in high-stage cells (UMUC3 and J82) increased by 8–10 times (Fig. 1A, B). Moreover, the time to induce ferroptosis is closely associated with tumor malignancy (Fig. S1A, B). ROS accumulation is the core event of triggering ferroptosis. We hypothesized that ROS production might vary among cell lines at different pathological stages. To test this hypothesis, we analyzed intracellular total ROS and lipid ROS levels using DCFH-DA and C11-BODIPY probes. Interestingly, intracellular total ROS and lipid ROS significantly increased in 5637 and T24 cells after RSL3 treatment, while only slightly elevated in UMUC3 and J82 cells (Fig. 2A–D and Fig. S1C). In line with this, malondialdehyde (MDA) levels, a primary product of lipid peroxidation, were correspondingly much more increased in 5637 and T24 compared to UMUC3 and J82 (Fig. S1D). Given that 3D oncosphere cells model may more accurately represent solid tumor responses to treatment, we treated BCa 3D cells model with RSL3. Similarly, low-stage BCa 3D models were sensitive to RSL3 therapy, while high-stage tumors exhibited significant resistance (Fig. 2E, F). Based on these in vitro findings, we further explored the therapeutic effect of RSL3-induced ferroptosis on BCa in vivo. As anticipated, we observed notable suppression of tumor growth in 5637 tumor-bearing mice treated by RSL3, while the treatment exhibited minimal effect on UMUC3 tumors (Fig. 2G–L and Fig. S1E, F). The level of PTGS2, a ferroptosis marker, increased by approximately 40-fold in 5637 tumor tissues, while only increasing by about 2-fold in corresponding UMUC3 tumor tissues (Fig. 2M, N). No significant changes in body weight and histomorphology of major organs were observed compared to the control group (Fig. S1G–I). Collectively, ferroptosis induction may represent a promising therapeutic strategy for low-stage BCa; however, its efficiency is significantly constrained in high-stage BCa due to ferroptosis resistance.

### Abnormal lipid metabolism may be implicated in ferroptosis escape in BCa

**Fig. 3:**
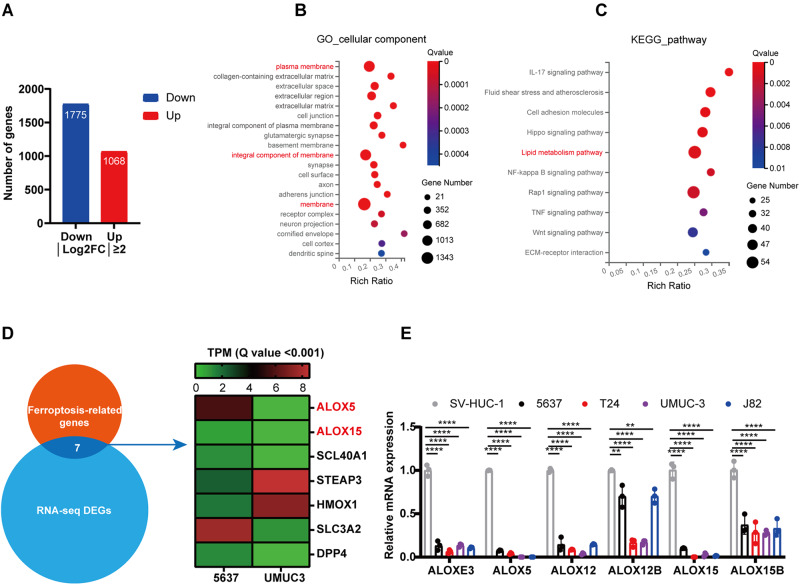
Abnormal lipid metabolism may be implicated in ferroptosis escape in BCa. **A** Differentially expressed genes (DEGs) of low-stage BCa cells (5637) and High-stage BCa cells (UMUC3). **B**, **C** Gene Ontology (GO) cellular component and KEGG pathway analyses were performed on the DEGs of 5637 and UMUC3 cells. **D** The ferroptosis-related genes in DEGs. **E** The relative expression levels of ALOXs in BCa cell lines. SV-HUC-1 cells as control. **p < 0.01, ****p < 0.0001. The data are represented as mean ± SD of three independent assays.

To delve deeper into the mechanism of ferroptosis escape in high-stage BCa cells, we performed RNA sequencing (RNA-seq) between low-stage (5637) and high-stage (UMUC3) BCa cells (Fig. S2A, B). We identified 2843 differentially expressed genes (DEGs) with log_2_FC > 2 between 5637 and UMUC3 cells (Fig. 3A). Gene Ontology (GO) analysis and Kyoto Encyclopedia of Genes and Genomes (KEGG) pathway enrichment analysis indicated that these DEGs were involved in impaired plasma membrane and abnormal lipid metabolism (Fig. 3B, C). Among the DEGs, seven ferroptosis-related genes were identified, including lipoxygenases (ALOX5 and ALOX15) that are associated with lipid metabolism (Fig. 3D). The mammalian lipoxygenase family (ALOXs) contains six isoforms, including ALOXE3, ALOX5, ALOX12, ALOX12B, ALOX15 and ALOX15B. To ascertain whether ALOXs mediated ferroptosis escape in high-stage BCa cells, we first examined the expression patterns of these genes in different pathological stage BCa cell lines using qRT-PCR and Western blotting (WB) assay. As depicted in Fig. 3E and Fig. S2C, the mRNA and protein expression levels of ALOXs were remarkably reduced in all BCa cell lines, except for ALOXE3 and ALOX12B proteins. The expression patterns of ALOX5 and ALOX12 were consistent with the sensitivity of ferroptosis induction in BCa cell lines. Together, these findings suggest that dysfunction of lipoxygenase may be a key factor mediating ferroptosis escape in BCa.

### The level of ALOX5 affects the sensitivity of BCa cells to ferroptosis in vitro

**Fig. 4:**
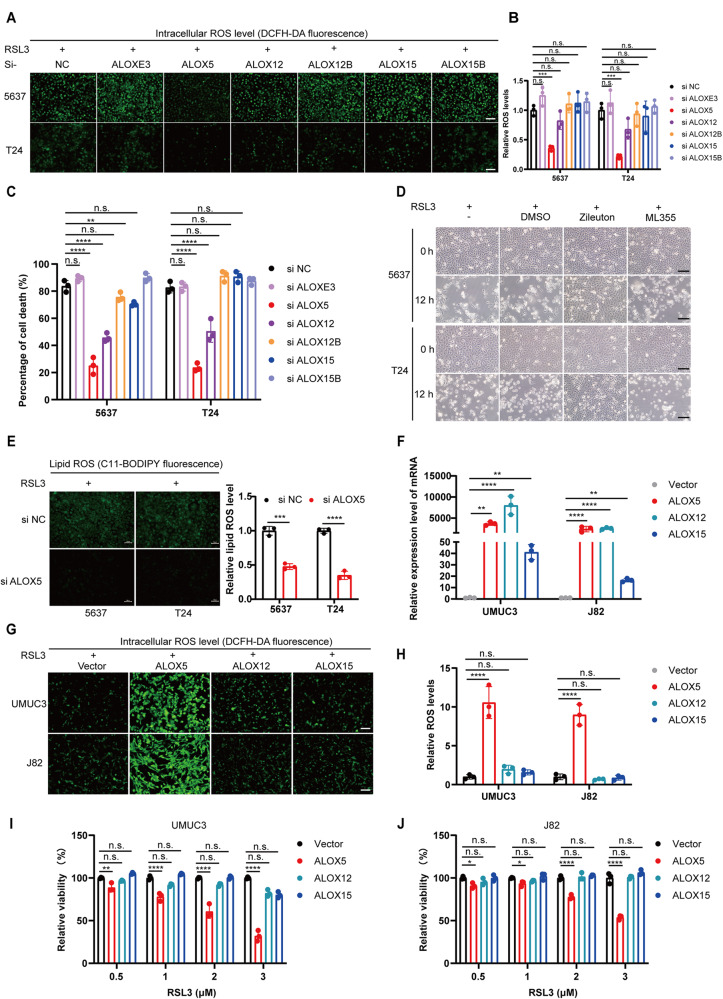
The level of ALOX5 affects the sensitivity of BCa cells to ferroptosis in vitro. **A** Intracellular total ROS levels were measured using the DCFH-DA probe in 5637 and T24 cells transfected with ALOXs siRNA. Scale bars, 100 μm. **B** The fluorescence intensity was detected using a fluorescence microplate reader. **C** BCa cells transfected with ALOXs siRNA were treated with RSL3 for 24 h, and the cell death ratio was detected using trypan blue at a concentration of 0.4%. **D** Representative images of BCa cells treated with RSL3 (1 μM) and specific inhibitors of ALOX5 (Zileuton, 40 μM) or ALOX12 (ML355, 4 μM) for 24 h. Scale bars, 100 μm. **E** BCa cells transfected with ALOX5 siRNA or control were treated with RSL3 (1 μM), and the lipid ROS level was measured using the C11-BODIPY probe. Scale bars, 50 μm. **F** The expression levels of ALOX5, ALOX12, and ALOX15 in UMUC3 and J82 cells after transfection with ALOXs overexpression plasmids. **G**, **H** BCa cells were transfected with ALOXs overexpression plasmids for 24 h and then treated with RSL3 (2 μM) for 24 h. The intracellular total ROS levels were measured using the DCFH-DA probe. The fluorescence intensity was assessed using a fluorescence microplate reader. Scale bars, 100 μm. **I**, **J** Viability (%) of BCa cells (UMUC3 and J82) after transfection with ALOXs overexpression plasmids for 24 h, and then treated with RSL3 (0.5, 1, 2, 3 μM) for 48 h. n.s., represent no significance, *p < 0.05, **p < 0.01, ***p < 0.001, ****p < 0.0001. The data are represented as mean ± SD of three independent assays.

To verify whether these ALOXs are involved in mediating resistance to ferroptosis induction in BCa, we conducted an RNAi-mediated loss-of-function screen experiment to determine whether the deficiency of any of these isoforms affects sensitivity to ferroptosis. As expected, the mRNA and protein level were decreased by corresponding siRNA (Fig. S3A–D). Interestingly, only the knockdown of ALOX5 significantly reduced intracellular ROS accumulation upon treatment with RSL3 (Fig. 4A, B). In line with this, ferroptosis was obviously attenuated by RNAi-mediated impairment of ALOX5 and ALOX12 (Fig. 4C and Fig. S3E), with ALOX5 achieving the best effect. To further investigate which ALOX or a combination of ALOX5 and ALOX12 determined the sensitivity of BCa to trigger ferrroptosis, we performed a pharmacological inhibition assay. We observed that ferroptosis was specifically blocked by Zileuton (a specific inhibitor of ALOX5), but not by ML355 (a specific inhibitor of ALOX12), indicating that ALOX5 is the crucial gene in mediating ferroptosis in BCa (Fig. 4D). Again, the lipid ROS level was markedly restrained by eliminating ALOX5 in BCa cells (Fig. 4E).

Subsequently, to further explore whether exogenous overexpression of ALOX5 reverse the resistance of high pathological stage BCa cells to ferroptosis induction, we transfected overexpression plasmids into UMUC3 and J82 cells. qRT-PCR and WB assay confirmed that the expression level of ALOXs mRNA and protein were successfully elevated (Fig. 4F and Fig. S4A). Indeed, the intracellular total ROS and lipid ROS dramatically accumulated in UMUC3 and J82 cells after overexpressing ALOX5 compared to control groups (Fig. 4G, H and Fig. S4B). In line with this, we observed that overexpression of ALOX5 significantly sensitized the high-stage cells to RSL3-induced ferroptosis, whereas ALOX12 and ALOX15 did not have that effect (Fig. 4I, J and Fig. S4C, D). Moreover, the sensitization effect was dose and time-dependent on ALOX5 expression levels (Fig. S4E), and that effect could be eliminated by Zileuton (Fig. S4F). Taken together, these data suggest that ALOX5 is the crucial gene in mediating ferroptosis in BCa.

### ALOX5 Knockout increased ferroptosis resistance of BCa cells in vivo

**Fig. 5:**
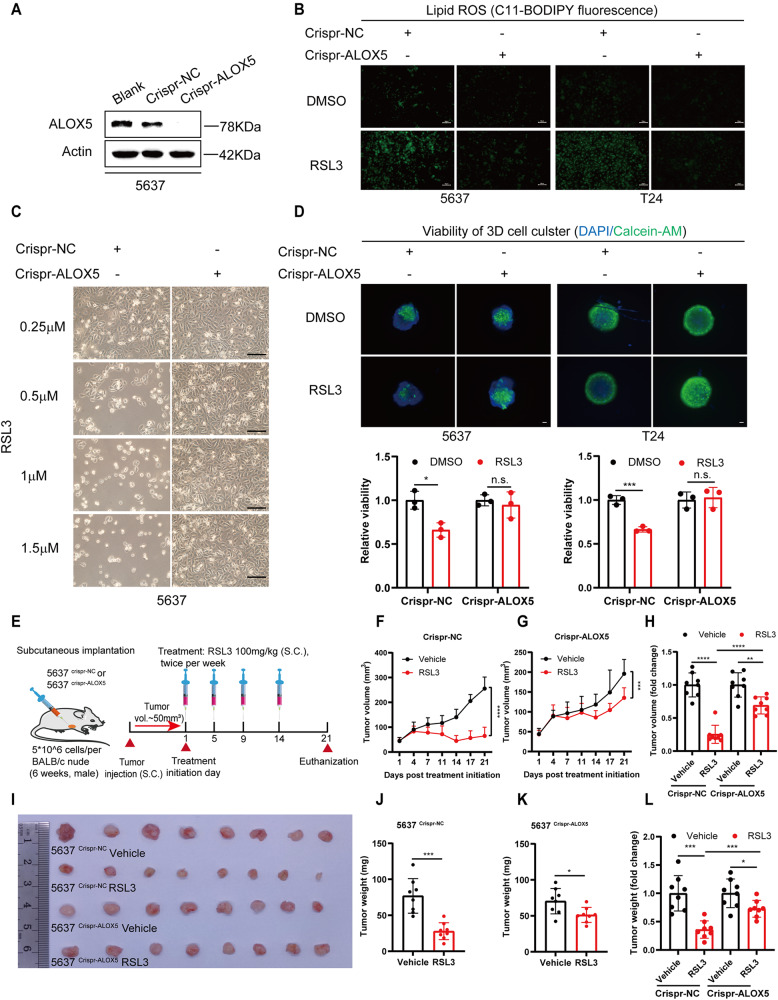
Knockout ALOX5 increased ferroptosis resistance of BCa cells in vivo. **A** Western blotting analyze the expression level of ALOX5 in 5637 ^crispr-ALOX5^ cells. **B** The lipid ROS levels were measured in BCa cells after ALOX5 knockout. RSL3, 1 μM. Scale bars, 50 μm. **C** Representative images of 5637 ^crispr-NC^ and 5637 ^crispr-ALOX5^ cells treated with RSL3 (0.25, 0.5, 1, 1.5 μM) for 24 h. Scale bars, 100 μm. **D** 3D spheroids of ALOX5 knockout 5637 cells were cultured with medium for three days and then treated with RSL3 (1 μM) for 48 h. Living cells were evaluated using Calcein-AM staining. Living cells were represented by green fluorescence, while DAPI staining indicated the nuclei (blue fluorescence). Scale bars, 200 μm. **E** The schematic diagram illustrates the construction of a xenograft tumor model and the dosage regimen, *n* = 8. **F**–**H** Growth curves of xenograft tumors after treatment with RSL3 (100 mg/kg) and vehicle. **I**–**L** Representative images of xenograft tumors after treatment with RSL3 and vehicle, and statistical analyses of tumor weight. n.s., represent no significance, *p < 0.05, **p < 0.01, ***p < 0.001, ****p < 0.0001. The data are represented as mean ± SD of three independent assays.

To further confirm the role of ALOX5 in mediating ferroptosis escape in BCa in vivo, we generated ALOX5-deficient 5637 and T24 cells using CRISPR/Cas9 technology (Fig. 5A and Fig. S5A). As expected, the lipid ROS levels were significantly reduced, and ferroptosis was inhibited in ALOX5 knockout BCa cells (Fig. 5B, C and Fig. S5B–D). The 3D oncosphere cells model also showed similar results, with the elimination of ALOX5 restrained RSL3-induced ferroptosis (Fig. 5D). In addition, the ferroptosis resistance produced by ALOX5 knockout could be restored by its re-expression (Fig. S5E).

Subsequently, we established xenograft tumors in BABL/c nude mice with ALOX5 knockout 5637 cells. When tumor volume reached 50~80mm^3^, mice were treated with RSL3 (100 mg/kg) or vehicle twice peer week for two weeks (Fig. 5E). Indeed, we observed that RSL3 significantly inhibited tumor growth in the Crispr-NC group. However, the suppression effect was significantly attenuated in the ALOX5 knockout group (Fig. 5F–L). Furthermore, the PTGS2 level was 5 times higher in the Crispr-NC group than that in the ALOX5 knockout group, while there was no significant difference in body weight between the two groups (Fig. S5F–J). Collectively, these data demonstrate that the absence of ALOX5 is crucial for ferroptosis escape in BCa.

### EGR1 transcriptionally activates ALOX5 expression

**Fig. 6:**
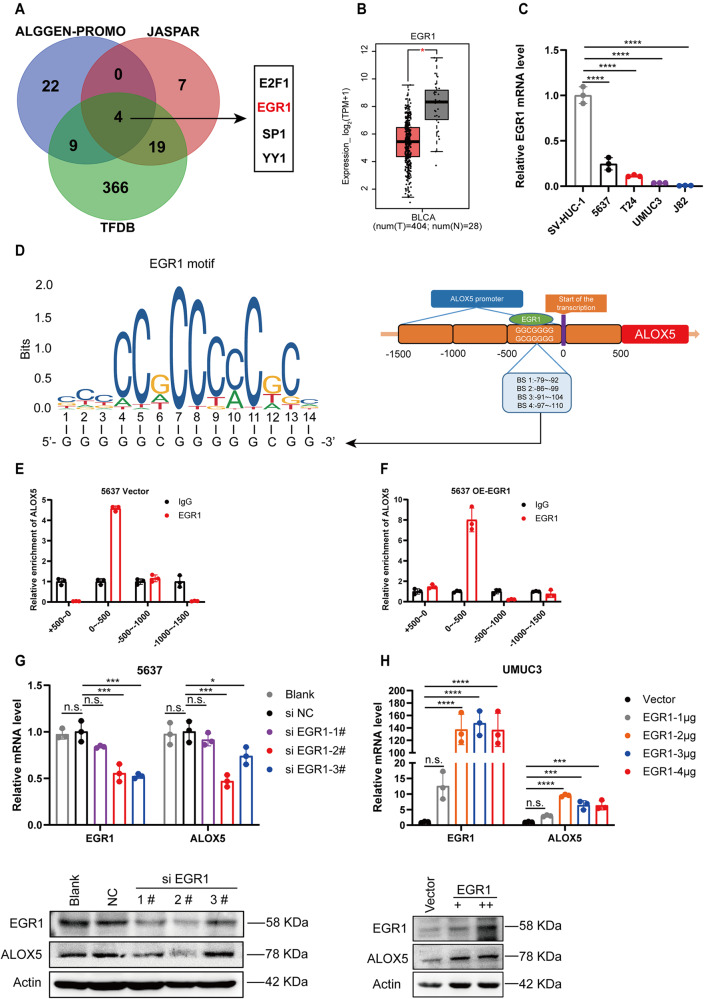
EGR1 transcriptionally activates ALOX5 expression. **A** Schematic diagram depicting the prediction of transcription factors using three databases. **B** The mRNA expression level of EGR1 in BCa database. **C** The mRNA expression level of EGR1 in BCa cell lines, SV-HUC-1 as control. **D** Schematic representation of the EGR1 binding site on the ALOX5 promoter and the corresponding EGR1 motif. **E**, **F** ChIP analysis demonstrating the binding of EGR1 to the ALOX5 promoter in 5637 cells with or without expression of EGR1. **G**, **H** Assessment of ALOX5 expression upon knockdown or overexpression of EGR1 in 5637 and UMUC3 cells. n.s., represent no significance, *p < 0.05, ***p < 0.001, ****p < 0.0001. The data are represented as mean ± SD of three independent assays.

The diminished expression of ALOX5 mRNA in BCa prompted us to hypothesize that it might be regulated at the transcriptional level. To test this hypothesis, we predicted transcription factors that could bind to the ALOX5 promoter region. We identified four candidate genes (E2F1, EGR1, SP1, and YY1) from three databases (Fig. 6A), among which E2F1 and EGR1 exhibited significant differential expressed in BCa datasets (Fig. 6B and Fig. S6A–C). We then assessed the expression levels of E2F1 and EGR1 in BCa cell lines and observed that the expression pattern of EGR1 was congruent with ALOX5 (Fig. 6C, Fig. 3E, and Fig. S6D, E). Therefore, we speculated that EGR1 might be an upstream transcription factor of ALOX5. With this mind, we predicted potential binding sites of EGR1 in the ALOX5 promoter region using the JASPAR website. As shown in Fig. 6D, there are four binding sites within the 500 bp upstream of the ALOX5 transcription start site (−79/−92, −86/−98, −91/−104, and −97/−110). Chromatin immunoprecipitation (ChIP) experiments confirmed that EGR1 could be enriched in the 0 to −500bp region of ALOX5 promoter, rather than the other three regions (+500 to 0, −500 to −1000 and −1000 to −1500) (Fig. 6E, F and Fig. S6F). Subsequently, we tested whether EGR1 influences the expression of ALOX5 in BCa cells. qRT-PCR and WB assays revealed that EGR1 knockdown markedly suppressed ALOX5 mRNA and protein levels, while EGR1 overexpression enhanced ALOX5 expression (Fig. 6G, H). Additionally, we observed that EGR1 knockdown or overexpression had the most significant impact on ALOX5 expression at 36 h (Fig. S6G–J). Collectively, these findings suggest that ALOX5 is transcriptionally regulated by EGR1.

### EGR1 mediates ferroptosis of BCa cells by transcriptional regulation of ALOX5

**Fig. 7:**
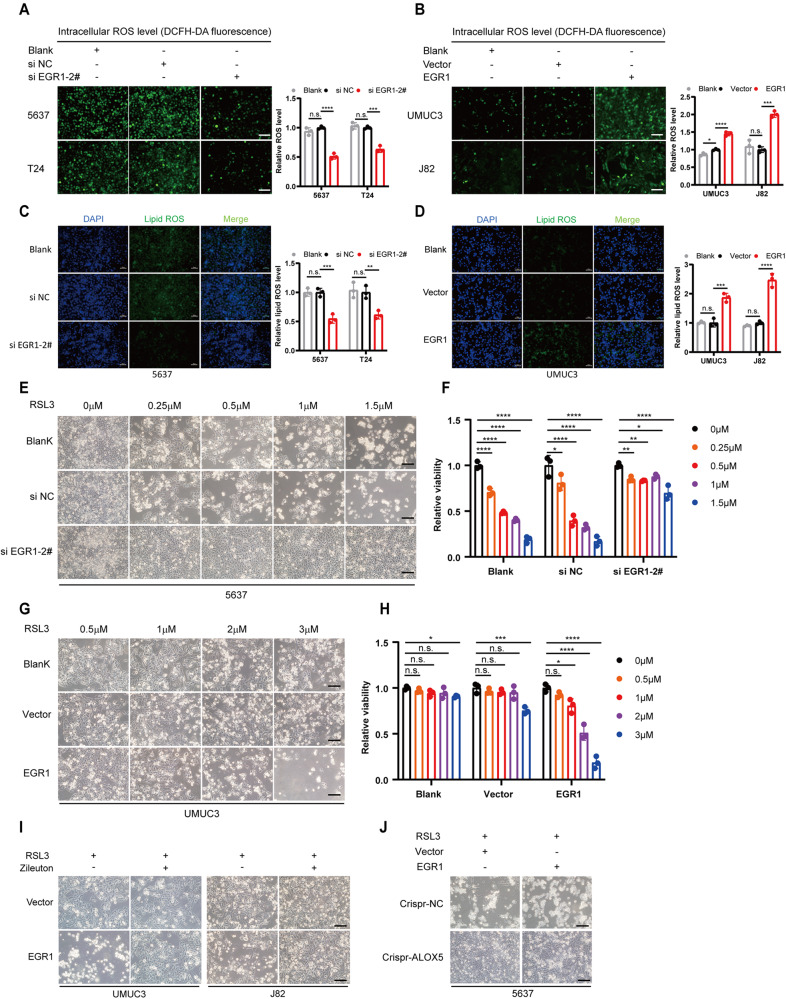
EGR1 mediates ferroptosis of BCa cells by transcriptional regulation of ALOX5. **A**, **C** Intracellular total ROS and lipid ROS levels were reduced in low-stage BCa cells following siRNA-mediated EGR1 deficiency. Scale bars, 50 μm. **B**, **D** Overexpression of EGR1 increased intracellular total ROS and lipid ROS levels in high-stage BCa cells. Scale bars, 50 μm. **E**, **F** Knockdown of EGR1 promoted resistance to RSL3-induced ferroptosis in 5637 cells. Cell viability was evaluated using the CCK-8 kit. Scale bars, 100 μm. **G**, **H** Overexpression of EGR1 increased the sensitivity of UMUC3 cells to ferroptosis. Cell viability was evaluated using the CCK-8 kit. Scale bars, 100 μm. **I** Zileuton rescue ferroptosis caused by overexpressing EGR1 in UMUC3 and J82 cells. UMUC3 treated with RSL3 (2 μM) for 48 h, and J82 treated with RSL3 (3 μM) for 48 h. Scale bars, 100 μm. **J** Overexpression of EGR1 in 5637 ^cirspr-ALOX5^ cells did not reverse the resistance of these cells to ferroptosis. Scale bars, 100 μm. n.s., represent no significance, *p < 0.05, **p < 0.01, ***p < 0.001, ****p < 0.0001. The data are represented as mean ± SD of three independent assays.

To investigate whether EGR1 plays a role in regulating ferroptosis in BCa cells, we initially measured the levels of intracellular ROS and lipid ROS in BCa cells with either knockdown or overexpression of EGR1. Intriguingly, EGR1 knockdown elicited a notable reduction in intracellular total ROS and lipid ROS in 5637 and T24 cells, respectively (Fig. 7A, C and Fig. S7A). Conversely, overexpression of EGR1 markedly augmented the accumulation of both ROS species in UMUC3 and J82 cells (Fig. 7B, D and Fig. S7B). In line with this, EGR1 depletion exhibited a pronounced restraint on ferroptosis in 5637 cells (Fig. 7E, F), whereas EGR1 overexpression partially reversed the resistance of UMUC3 cells to RSL3-induced ferroptosis, thereby increased their sensitivity to ferroptosis (Fig. 7G, H and Fig. S7C). Subsequently, we sought to elucidate whether EGR1 exerts its impact on ferroptosis through the EGR1-ALOX5 axis. To this end, we conducted a rescue experiment employing the ALOX5 inhibitor Zileuton. As depicted in Fig. 7I, the ferroptosis-sensitizing effect of EGR1 was abrogated by Zileuton. Furthermore, we overexpressed EGR1 in ALOX5 knockout cells. Interestingly, EGR1 overexpression did not restore ferroptosis sensitivity in ALOX5-deficient cells (Fig. 7J). Collectively, these findings suggest that EGR1 may sensitize BCa cells to ferroptosis by transcriptionally activating ALOX5.

### The expression pattern of ALOX5 in BCa clinical specimens was negatively associated with tumor progression and outcome

**Fig. 8:**
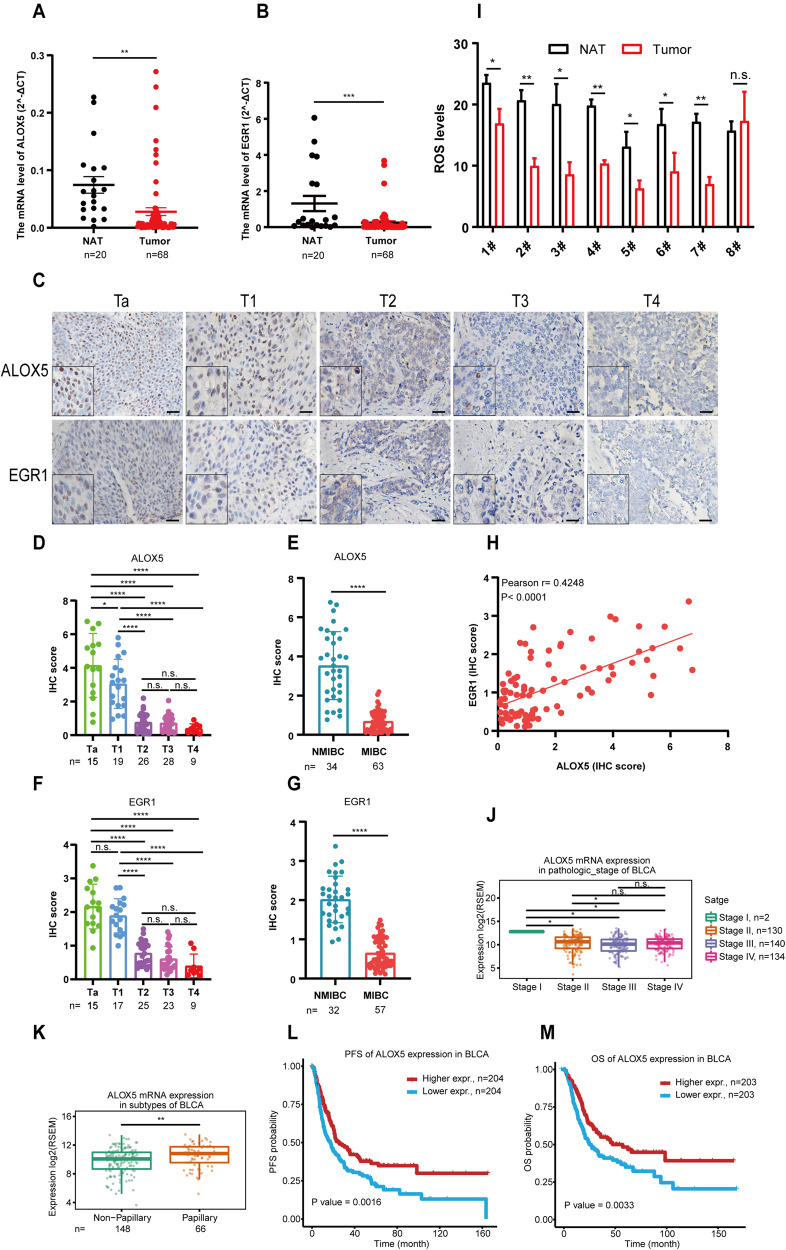
The expression pattern of ALOX5 in BCa clinical specimens was negatively associated with tumor progression and outcome. **A**, **B** The expression level of ALOX5 and EGR1 in BCa clinical specimens. NAT (normal adjacent tissue, *n* = 20). Tumor (*n* = 68). The data are represented as mean ± SEM. **C**–**G** Representative images of IHC staining in BCa clinical specimens, and statistical analyses of IHC score. Scale bars, 100 μm. **H** Pearson correlation analysis of ALOX5 and EGR1 IHC scores. **I** ROS level in BCa clinical specimens. **J**, **K** The expression level of ALOX5 in TCGA database. **L**, **M** Kaplan–Meier analysis of the correlation between ALOX5 expression and prognosis outcome. n.s., represent no significance, *p < 0.05, **p < 0.01, ***p < 0.001, ****p < 0.0001. The data are represented as mean ± SD.

The above results indicate that ALOX5 is a crucial regulator of ferroptosis in BCa. To validate the expression pattern of ALOX5 and its upstream transcription factor EGR1 in clinical specimens, we collected a cohort consisting of 20 pairs of BCa tissues and their corresponding adjacent normal tissues, as well as 48 BCa tissues from the department of urology specimen bank. qRT-PCR analysis revealed a significant decrease in the mRNA levels of ALOX5 and EGR1 in BCa tissues compared to normal tissues (Fig. 8A, B). Furthermore, IHC analysis of consecutive tissue sections from another cohort with different pathological stages demonstrated a consistent down-regulation of ALOX5 and EGR1, which was associated with advanced pathological stages (Fig. 8C–G). Importantly, Pearson correlation analysis showed a positive correlation between the expression levels of ALOX5 and EGR1 (Fig. 8H). In line with these findings, we observed a decrease in the level of ROS in the majority BCa cases (7/8) (Fig. 8I). Furthermore, our exploration of the TCGA database provided additional support for the close association between ALOX5 levels and the malignancy of BCa (Fig. 8J, K). Additionally, Kaplan-Meier survival analysis revealed a significant correlation between lower expression of ALOX5 and poor patient outcomes (Fig. 8L, M). Taken together, these clinical data confirm a strong association between ALOX5 and EGR1, highlighting the potential of ALOX5 as a promising prognostic indicator for BCa progression.

## Discussion and conclusion

Ferroptosis is an iron-dependent form of regulated cell death driven by the accumulation of lethal lipid peroxides. Since massive proliferation of tumor cells are generally accompanied by increased ROS and unstable iron overload, tumor cells are theoretically more vulnerable to ferroptosis. Based on this, the potential of ferroptosis-based therapies has attracted intense attention, particularly for cases resistant to conventional treatments. Ghoochani et al. demonstrated that ferroptosis inducers (FINs) inhibit the progression of various variants of prostate cancer, including androgen-sensitive and castration-resistant prostate cancer. Moreover, FINs combined with second-generation anti-androgen had significantly synergistic anti-tumor effects [26]. Likewise, Roh et al. observed that induced ferroptosis could inhibit the growth of cisplatin-resistant head and neck tumor cells in vivo [38]. Yet, during tumor progression, cancer cells have developed various strategies, such as enhancing antioxidant capacity or inhibiting lipoxygenase activity to protect themselves against ferroptosis [35, 39–41]. Therefore, elucidating the ferroptosis escape mechanism is essential for developing new strategies based on ferroptosis-induced therapy. To date, the therapeutic potential of induced ferroptosis in BCa remains largely unknown, and whether a ferroptosis escape mechanism exists in BCa needs further investigation.

In this study, we found that low-stage BCa cells are highly sensitive to RSL3-induced ferroptosis in vitro and in vivo, whereas high-stage BCa cells exhibited 8–10-fold resistance than low-stage BCa cells. This finding suggests that ferroptosis induction may be a promising treatment option for early BCa, but not suitable for advanced tumors. Meanwhile, tumor development may be accompanied by a special mechanism of ferroptosis escape. Using RNA-seq technology and RNAi-mediated loss-of-function screen assays, we demonstrated that ALOX5-mediated oxidative dysfunction plays a critical role in BCa acquiring ferroptosis escape. Moreover, ALOX5 expression was markedly decreased in BCa clinical specimens and closely associated with tumor malignancy and poor post-operation outcomes. These results are consistent with previous research evidence [42–44]. Therefore, ALOX5 deficiency is a key factor in mediating ferroptosis escape in BCa progression. Furthermore, ALOX5 may serve as a potential biomarker to distinguish the population that would benefit from ferroptosis induction therapy.

In this work, we observed consistent decreases in ALOX5 mRNA and protein levels in BCa cell lines and tissues, leading us to speculate that ALOX5 may be affected at the transcriptional level. Using the transcription factor prediction database, we found that multiple transcription factor could bind to the ALOX5 promoter region, among which E2F1, EGR1, SP1, and YY1 are overlapped genes by three software. However, only the expression pattern of EGR1 was consistent with ALOX5. Previous studies reported that the core binding sequence, GCGGGGGCG, of EGR1 is highly conserved in various cells, including HeLa cell, mouse and human fibroblasts [45]. Surprisingly, we observed that the 500 bp upstream region of ALOX5 promoter contains four consecutive sites that perfectly match the EGR1 core binding sequence. This finding indicates that EGR1 may have a strong affinity with the ALOX5 promoter and regulates its expression. Indeed, using ChIP assays, we further confirmed that EGR1 binds to the 500 bp promoter region upstream of the ALOX5 transcription start site and activates ALOX5 expression in BCa cells. In addition to being regulated by transcription factors, whether there are other transcriptional regulation modes, such as DNA methylation, remains unclear. As a classical transcription factor, EGR1 has multiple roles in tumors, involving cell processes including proliferation, differentiation, invasion, and apoptosis. Functional experiments on EGR1 revealed that increased EGR1 expression was associated with the malignancy of prostate cancer and gastric cancer [46, 47]. However, our data displayed that the EGR1 levels are significantly decreased in BCa cell lines and clinical specimens compared to normal controls. Meanwhile, EGR1 levels are negatively correlated with the pathological stage of BCa. These opposite biological functions may be determined by tissue specificity, similar to the function of RBMS1 [48, 49]. Our results validate the role of ALOX5 in the ferroptosis escape of BCa. Resensitizing high-stage BCa to ferroptosis is the key to determining the therapeutic strategy of ferroptosis induction. We found that exogenous overexpression of ALOX5 or EGR1 markedly attenuated the resistance of high-stage BCa cells to ferroptosis induction. In addition, traditional anti-tumor regents, including cislpatin and anti-PD-L1 antibody also have the function of promoting ferroptosis [29, 50], while ferroptosis resistance may be a crucial factor in treatment failure. Therefore, supplementing ALOX5 to relieve the resistance of high-stage BCa to ferroptosis may contribute to sensitizing traditional chemotherapy and immunotherapy.

In conclusion, this study highlights the potential of ferroptosis induction as a promising therapeutic approach for low-stage BCa, while identifying its limitations in treating advanced tumors. Most importantly, our work uncovers a novel mechanism for BCa ferroptosis escape and proposes that ALOX5 may be a valuable therapeutic target and prognostic biomarker in BCa treatment.

## Materials and methods

### Cell culture

The cell lines SV-HUC-1, T24, 5637, UMUC3, and J82 were obtained from the Shanghai Institute of Cell Biology at the Chinese Academy of Sciences (Shanghai, China). Prior to their use, all cell lines were authenticated by short tandem repeat profiling and confirmed the absence of mycoplasma contamination. All cells were cultured in a humidified condition at 37 °C with 5% CO_2_. All cells were cultured in a suitable medium contained 10% FBS (Gibco), 100 U/mL penicillin, and 100 μg/mL streptomycin (Gibco). The medium was replenished every other day to maintain optimal cell growth and viability.

### Patient specimens

Patient tissue specimens of bladder cancer and adjacent non-cancer tissues were acquired from the Affiliated Drum Tower Hospital of Nanjing University, School of Medicine (Nanjing, China). The study protocol involving the use of patient specimens was approved by the Ethics Committee of The Affiliated Drum Tower Hospital of Nanjing University, School of Medicine (Approval No. 2021-394-01). All clinical samples used in this study were collected with the informed consent of patients. The collection and use of patient specimens were conducted in accordance with the guidelines outlined in the Helsinki Declaration, ensuring the ethical treatment of human subjects.

### Reagents and antibodies

The following chemical inhibitors were used in this study: Z-VAD-FMK (apoptosis inhibitor, A1902), Necrostatin-1 (necroptosis inhibitor, A4213), and 3-methyladenine (3-MA, autophagy inhibitor, A8353) were purchased from APExBIO (USA). Ferrostatin-1 (ferroptosis inhibitor, HY-100579) and Deferoxamine (DFO, ferroptosis inhibitor, HY-B1625) were obtained from MedChemExpress (MCE, USA). For in vitro experiments, (1S, 3R)-RSL3 (RSL3, ferroptosis inducer, HY-100218A) was purchased from MedChemExpress (MCE, USA). For in vivo experiments, (1S, 3 R)-RSL3 (RSL3, ferroptosis inducer, CSN17581) was obtained from CSNpharm (USA). ML355 (ALOX12 inhibitor, HY-12341) and Zileuton (ALOX5 inhibitor, HY-14164) were purchased from MedChemExpress (MCE, USA). All the above-mentioned drugs were dissolved according to the manufacturer’s instructions and used at different doses as per the experimental requirements. The specific details can be found in the figure legends.

The following antibodies were used for Western blotting experiments: Anti-ALOXE3 (A8215, 1:1000) and anti-ALOX12B (A16210, 1: 750) were purchased from ABclonal technology (Wuhan, China). Anti-ALOX5 (sc-136195, 1:750), anti-ALOX12 (sc-365194, 1:750), anti-ALOX15 (sc-133085, 1:750) and anti-ALOX15B, (sc-271290, 1:500) were obtained from Santa Cruz Biotechnology (USA). Anti-ALOX5 (T58019S,1:1500), anti-EGR1 (T57177s, 1:30000), anti-E2F1(T50586, 1:1500), anti-Actin (M20011F, 1:5000), anti-GAPDH (M20006S, 1:5000), goat anti-mouse IgG-HRP (M21001S, 1:5000) and goat anti-rabit IgG-HRP (M21002S, 1:5000) were purchased from Abmart (China). The following antibody dilution concentrations were used for immunohistochemistry experiments: Anti-ALOX5 (T58019S, 1:400) and anti-EGR1 (T57177s, 1:400), purchased from Abmart (China).

### Cell viability and cell death assays

For the cell viability experiment, the following procedure was followed: 5 × 10^3^ cells were inoculated into each well of a 96-well plate and incubated under appropriate conditions for 24 h. After that, the cells were treated with different reagents for the indicated time. The medium with reagents was then replaced with 100 μl of fresh medium containing 10 μl of Cell Counting Kit-8 (CCK-8, A311-2 Vazyme, China). The 96-well plate was further incubated for 2 h at 37 °C. Following the incubation, the absorbance of each well was measured at a wavelength of 450 nm using a fluorescence microplate reader (SpectraMax M2, USA). The results were normalized to the negative control and expressed as a percentage.

For the cell death assay, the following procedure was followed: 5 × 10^5^ cells were seeded into each well of a 12-well plate and incubated under appropriate conditions for 24 h. The cells were then exposed to various reagents to induce cell death. At the appropriate time point, the cells were collected. The collected cells were stained with trypan blue at a concentration of 0.4% for 3 min. Subsequently, the percentage of cell death was determined using an automated cell counter (6479, Corning, USA).

### Colony formation assay

The colony formation assay was performed to assess the proliferation capability of single cells. The procedure was as follows: Cells were pre-treated with various drugs or transfected as required. The cells were then seeded in 6-well plates at a density of 2000 cells per well. The cells were cultured in a suitable incubator for a period of 7–12 days. Every three days, the medium in each well was replaced with fresh medium with or without the respective drug. After the designated incubation period, the colony masses were fixed with 4% paraformaldehyde. The fixed colonies were stained with 0.5% crystal violet for 20 min. Subsequently, the number of colonies was counted using image analysis software such as ImageJ.

### Transwell invasion assay

The transwell invasion assay was conducted to evaluate the invasion capability of cells using a transwell chamber with pre-coated matrigel. The procedure is as follows: Pre-treated cells, resuspended in fresh medium without fetal bovine serum (FBS), were seeded in the upper chamber of a transwell chamber (5 × 10^4^ cells per well, 200 μl). In the lower chamber, 500 μl of fresh medium containing 10% FBS, with or without the drug of interest, was added. The transwell chamber was then incubated under suitable conditions for the indicated time period, according to the invasion capabilities of the specific cells being studied. After the incubation period, the cells that passed through the membrane and invaded into the lower chamber were fixed with 4% paraformaldehyde. The invaded cells were stained with 0.5% crystal violet for 20 min. Subsequently, the invaded cells were captured using an electron microscope (Olympus, Germany), and three randomly selected fields were used to calculate the invasion rate.

### Wound healing assay

The wound healing assay was performed to evaluate the migratory capability of bladder cancer cells following pre-treatment with drugs or transfection. The procedure is as follows: Cells were inoculated into a 6-well plate and allowed to reach 90% confluence. Using a 200 μl pipette tip, a wound (scratch) was created in each well, carefully making a straight line across the cell monolayer. The migration process of the cells was recorded at 0, 12, and 24 h using an electron microscope (Olympus, Germany) or a suitable imaging system. The migratory area, representing the closure of the wound, was quantified using image analysis software such as ImageJ.

### Three-dimensional (3D) cell viability assay

To better simulate the cellular environment in vivo compared to traditional 2D cell culture, a 3D cell model was constructed using an ultra-low absorption plate (U-bottom, Lv-ULA002-96UW, Liver Biotechnology, China). The procedure is as follows: 2 × 10^3^ cells were seeded in each well of a 96-well ultra-low absorption plate, with 10% fetal bovine serum (FBS) in the culture medium. The plate was then incubated at 37 °C and 5% CO_2_ for a period of 3–5 days to allow the cells to form a 3D structure. After the initial incubation period, the culture medium was exchanged with fresh medium containing the desired reagents, and the plate was further incubated for 48 h. To evaluate the viability of the live cells, the cells were stained with Calcein-AM (C2012, Beyotime, China) according to the manufacturer’s instructions. The cell viability was measured using a Cell Counting Kit-8 (CCK-8) kit (A311-2, Vazyme, China) or a suitable assay kit.

### Intracellular ROS measurements

Intracellular reactive oxygen species (ROS) levels were measured using the following protocol: Cells, either treated with transfection or untreated, were seeded in 12-well plates and cultured for 24 h. Reagents were added to the culture medium to induce ROS accumulation for an appropriate period of time, as determined by the experimental requirements. The initial medium was then replaced with 1 mL of fresh serum-free medium containing a suitable concentration of the DCFH-DA fluorescent probe (YI FEI XUE, Nanjing, China), and the cells were incubated for 20 min at 37 °C. After the incubation period, the cells were washed three times with serum-free medium to remove residual DCFH-DA fluorescent probe. The green fluorescence emitted by the intracellular ROS was visualized using a fluorescence microscope (Olympus, Germany). The intensity of ROS in the cells was quantitatively analyzed using a fluorescence microplate reader (spectra MAX M2, USA).

### Lipid peroxidation assay

Lipid peroxidation, a crucial indicator of ferroptosis, was measured using the following method. In brief, 5 × 10^5^ cells were inoculated into a 12-well plate and treated with the test reagents for the indicated time on the next day. Then, the initial medium was replaced with 1 mL fresh FBS-free medium contained C11-BODIPY 581/591 (2 μM, Invitrogen, D3861) and the cells returned to incubate for 30 min. Subsequently, the cells washed with FBS-free medium for 3 times to remove the residue C11-BODIPY 581/591 fluorescent probe. The relative strength of green fluorescence was measured by a fluorescence microscope (BX53, Olympus, Germany). The quantitative analysis of the fluorescence intensity was evaluated by image J software.

### MDA assay

The level of malondialdehyde (MDA), a compound formed during the decomposition of lipid peroxides and an indicator of lipid peroxidation, was measured using an MDA assay kit (M496, DOJINDO, Japan) following the manufacturer’s instructions. The relative concentration of MDA was measured using a fluorescence microplate reader (spectra MAX M2, USA).

### RNA-sequencing and analysis

The transcriptome sequencing and analysis were performed as follows: Total RNA from 5637 and UMUC3 cells was extracted using Trizol reagent (390206, Life Technology, USA). The concentration and quality of the RNA samples were assessed using a Nanodrop spectrophotometer (Thermo Fisher Scientific, USA) to ensure high-quality RNA for subsequent analysis. Library construction and sequencing of the RNA samples were carried out by BGI (Shenzhen, China). The differential expressed genes with log2FC ≥ 2 were screened for further GO and KEGG analysis.

### Quantitative real-time polymerase chain reaction (qRT-PCR)

Total RNA from cells and tissues was isolated using Trizol reagent (Life Technology, USA) following the manufacturer’s instructions. The concentration and quality of the RNA samples were assessed using a Nanodrop spectrophotometer (ThermoFisher Scientific, USA). For reverse transcription polymerase chain reaction (RT-PCR), 1 μg of RNA was reverse transcribed into complementary DNA (cDNA) using an RT reagent kit (R323, Vazyme, China). The cDNA served as the template for quantitative PCR (qPCR). Quantitative PCR was performed using the ChamQ Universal SYBR qPCR Master Mix (Q711, Vazyme, China) in a 7300 real-time PCR system (ABI, Japan). SYBR Green dye was used to detect the amplification of the target DNA during each PCR cycle, and the fluorescence signal was measured in real-time. The primers were used in this study are as follows:

ALOXE3 forward 5′-CTACCACTACCGAGACGACG-3′, ALOXE3 reverse 5′-AGGAACGCCTGAGCAAAA-3′; ALOX5 forward 5′-CCTATGCCTCCCTGTGCT-3′, ALOX5 reverse TGGTCGCCCTCGTAGTAGA-3′; ALOX12 forward 5′-CCAAAGGGATGACATAGTGAA-3′, ALOX12 reverse 5′-GGTGAGGAAATGGCAGAGTT-3′; ALOX12B forward 5′-TCTGAGTGGGACTGGCTGTTGG-3′, ALOX12B reverse 5′-AGAGACCTCCCTTGTTGAGAAG-3′; ALOX15 forward 5′-CCTATGCCTCCCTGTGCT-3′, ALOX15 reverse 5′-TGGTCGCCCTCGTAGTAGA-3′; ALOX15B forward 5′-GATCTTCAACTTCCGGAGGAC-3′, ALOX15B reverse 5′-ACTGGGAGGCGAAGAAGG-3′; PTGS2 forward 5′-CTGATGATTGCCCGACTCCC-3′, PTGS2 reverse 5′-TCGTAGTCGAGGTCATAGTTC-3′; E2F1 forward 5′-GGGGGAGAAGTCACGCTATG-3′, E2F1 reverse 5′-AAACATCGATCGGGCCTTGT-3′; EGR1 forward 5′-CAGCACCTTCAACCCTCAG-3′, EGR1 reverse 5′-CACAAGGTGTTGCCACTGTT-3′; GAPDH forward 5′-GGAGCGAGATCCCTCCAAAAT-3′, GAPDH reverse 5′-GGCTGTTGTCATACTTCTCATGG-3′.

### Western blot (WB) assays

For Western blot (WB) assays, the following procedure was followed: Proteins were extracted from cells or tissues using RIPA lysis buffer (Beyotime, China) containing protease inhibitors (Beyotime, China) and phosphatase inhibitors (Beyotime, China). The protein concentration was determined using a BCA protein assay kit (ThermoFisher Scientific, USA). The extracted proteins were separated by 10% SDS-PAGE (sodium dodecyl sulfate-polyacrylamide gel electrophoresis) based on their molecular weight. The separated proteins were transferred from the gel to a PVDF membrane (polyvinylidene fluoride membrane) using appropriate transfer conditions. The PVDF membrane was blocked in 5% skim milk for 1 h at room temperature. The membrane was incubated with primary antibodies specific to the target protein of interest. This step was performed overnight at 4 °C to allow for antibody-antigen binding. After washing off unbound primary antibodies, the membrane was incubated with corresponding secondary antibodies. The protein bands on the membrane were visualized using an imaging system such as Tanon (5200, Shanghai, China).

### si-RNA synthesized, over-expression plasmids construction

si-RNA was synthesized to transiently knock down the expression level of the target gene in vitro cells. si-ALOX5, si-ALOX12 and si-ALOX15 were purchased from RIBOBIO (Guangzhou, China), while si-ALOXE3, si-ALOX12B, si-ALOX15B and si-EGR1 were obtained from TSINGKE (Beijing, China). The si-RNA targeting sequences used were as follows:

si-ALOX5-1#: GATTCACCATTGCAATCAA;

si-ALOX5-2#: GCAACACCGACGTAAAGAA ;

si-ALOX12-1#: GGAGTTTGATCATGACGTT;

si-ALOX12-2#: GCAAACGGCACCTCCTTAA;

si-ALOX15-1#: TCGTGAGTCTCCACTATAA;

si-ALOX15-2#: GCCATCTCATGGCATCTGA;

si-ALOXE3: GACCTTCACGACAAAGTAT;

si-ALOX12B: CGCTATGCGGAGTTCTACA;

si-ALOX15B: CAATGACCCTGCTATACCA;

si-EGR1-1#: GCGGCAGAAGGACAAGAAA;

si-EGR1-2#: GCCTAGTGAGCATGACCAA;

si-EGR1-3#: CCATGGACAACTACCCTAA.

On the other hand, over-expression plasmids were used to transiently increase the expression level of target genes in vitro cells. ALOX5, ALOX12, and ALOX15 plasmids were purchased from Corues Biotechnology (Nanjing, China), while EGR1 and ALOX5 lentivirus vector plasmids were obtained from GenScript (Nanjing, China). All constructs were confirmed by DNA sequencing to ensure their accuracy.

### Cell transfection assay

For transfection assay, cells were seeded into 6-well plates and reached 70-80% confluence. After that, siRNA or over-expression plasmids were transfected into cells using GenEscort®II transfenction reagent (Wisegen Biotechnology Corporation, Nanjing, China) according to the manufacturer’s instruction. The efficiency of transfection was confirmed by qRT-PCR and Western blot assays.

### CRISPR-Cas9 assay

To generate ALOX5 CRISPR-Cas9 knockout 5637 cells, a lentivirus carrying ALOX5 single guide RNA (sgRNA) was constructed by GenScript (Nanjing, China). The lentivirus containing ALOX5 sgRNA was used to infect the cells. After infection, the cells were subjected to puromycin selection with increasing concentrations (1 μg, 3 μg, 5 μg, 10 μg) for a period of 2 weeks. During the puromycin selection process, cells that do not carry the ALOX5 knockout were eliminated, while the cells with successful knockout were able to survive and proliferate. The efficiency of CRISPR-Cas9 knockout was assessed by performing a western blot assay. The target sgRNA sequence of ALOX5 was: CACGUCGUAUGAAUCCACCU. This specific sequence guides the Cas9 enzyme to the target site in the ALOX5 gene, facilitating the knockout of the gene.

### Chromatin immunoprecipitation qPCR (ChIP-qPCR) analysis

ChIP experiment was performed following the instructions provided by the manufacturer (Cell Signaling Technology, CST, USA). In briefly, 5637 cells with or without over-expressed EGR1 were fixed with 1% formaldehyde for 15 min and then terminated cross-linking using 0.12 M glycine for 10 min at room temperature. Cells nuclear precipitation were extracted using TNT buffer and the chromatin DNA was then fragmented into fragments ranging from 200 to 1000 bp by sonication. 5–10 μg chromatin fragments were incubated with specific EGR1 antibody or IgG at 4 °C overnight. The immunocomplexes were captured using Dynabeads^TM^ protein G (Invitrogen, USA), which specifically binds to the antibody. The beads were then washed to remove nonspecific binding and contaminants. The immunocomplexes were subjected to a series of treatments to reverse the cross-linking, resulting in the release of DNA fragments. These fragments contained the enriched promoter sequences of ALOX5, specifically bound by EGR1. Finally, the enrichment of the ALOX5 promoter sequences was measured using quantitative PCR. The following primer pairs were used for qPCR amplification of specific regions within the ALOX5 promoter:

Primer 1 (0–500 bps): Forward 5′-AAGCGTTGGTGAGAATGT-3′, Reverse 5′-TGTATTGATCTCGGCTAAGT-3′;

Primer 2 (0 to −500bps): Forward 5′-GTGGCACTGAGAACTTGG-3′, Reverse 5′-CTCTCCTGAATTGCTTCCA-3′;

Primer 3 (−500 to −1000bps): Forward 5′-GCACTGACGACTACATCTA-3′, Reverse 5′-GTAGAAGGGCTTGTCCAG-3′,

Primer 4 (−1000 to −1500bps): Forward 5′-GGAGAGGCAGATGAGTCA-3′, Reverse 5′-CCAGACAGACGGTAGAGT-3′.

### Immunohistochemical staining

The paraffin-embedded sections of patient tumor tissues underwent dewaxing, antigen repair, and then incubated with specific primary antibodies (anti-ALOX5, anti-EGR1) at 4 °C overnight. Subsequently, the sections were incubated with a secondary antibody for 1 h at room temperature and then measured using DAB reagent. The intensity of positive results was analyzed using H-score as follows: 0 (negative), 1 (weak), 2 (moderate), 3 (high). The proportion of positive cell stained were assessed as follows: 0 (none), 1 (1%-25%), 2 (26%-50%), 3 (51%-75%), 4 (>75%).

### Bioinformatics analysis

The RNA-seq data of bladder cancer tissues were obtained from The Cancer Genome Atlas (TCGA) official website. The Overall survival (OS) and progression-free survival (PFS) data were obtained from the Kaplan-Meier plotter (https://kmplot.com/analysis/). The potential transcription factor binding sites in the ALOX5 promoter region were predicted using JASPAR 2022 (https://jaspar.genereg.net/).

### Animal models experiments

All animal experiments in this study were approved by the Animal Ethical and Welfare Committee of Nanjing University (IACUC-2302004), and followed the guidelines of the Institutional Animal Care and Use Committee.

Five-week-old BALB/c nude mice (male, 16-18 g, SPF grade) were purchased from GemPharmatech (Nanjing, China). For the subcutaneous xenograft model, bladder cancer cells (5637, UMUC3, 5637^Crispr-NC^, 5637^Crispr-ALOX5^) were suspended in 100 μl PBS mixed with matrigel (1:1 ratio) and injected into the armpit of nude mice at a concentration of 5 × 10^6^ cells/mice. When tumor volume reached 50 to 80 mm^3^, the mice were randomly divided into two groups (no blinding was done) and then treated with intratumor injections of RSL3 (100 mg/kg) twice per week for 2 weeks. Tumor volume (V = length × width^2^ × 0.52) and body weight were monitored every 3 days during the treatment period.

### Statistical analysis

The sample size was chosen based on the need for statistical power. Statistical analysis was conducted using GraphPad Prism 9.0 software. All cell experiments were repeated at least three times. The data are presented as the mean ± SEM (standard error of the mean) unless stated otherwise. Student’s *t* test was used for comparisons between two groups, while one-way ANOVA and two-way ANOVA were employed for multiple-group comparisons. *p*-value less than 0.05 was considered statistically significant. The significance levels were denoted as follows: *p < 0.05, **p < 0.01, ***p < 0.001, ****p < 0.0001, and “n.s.” indicated no significant difference. The letter “*n*” represents the number of replicates.

### Supplementary information

Figure S1

Figure S2

Figure S3

Figure S4

Figure S5

Figure S6

Figure S7

Supplementary Figures legend

Original Western blot data

Reproducibility checklist

## Data Availability

The datasets used and/or analyzed during this study are available from the corresponding author on reasonable request.
