# Supplementary figures and images for: ALOX5 deficiency contributes to bladder cancer progression by mediating ferroptosis escape
Source: Cell Death Dis. 2023 Dec 7;14(12):800. doi: 10.1038/s41419-023-06333-7 (PMC10703795; doi:10.1038/s41419-023-06333-7)

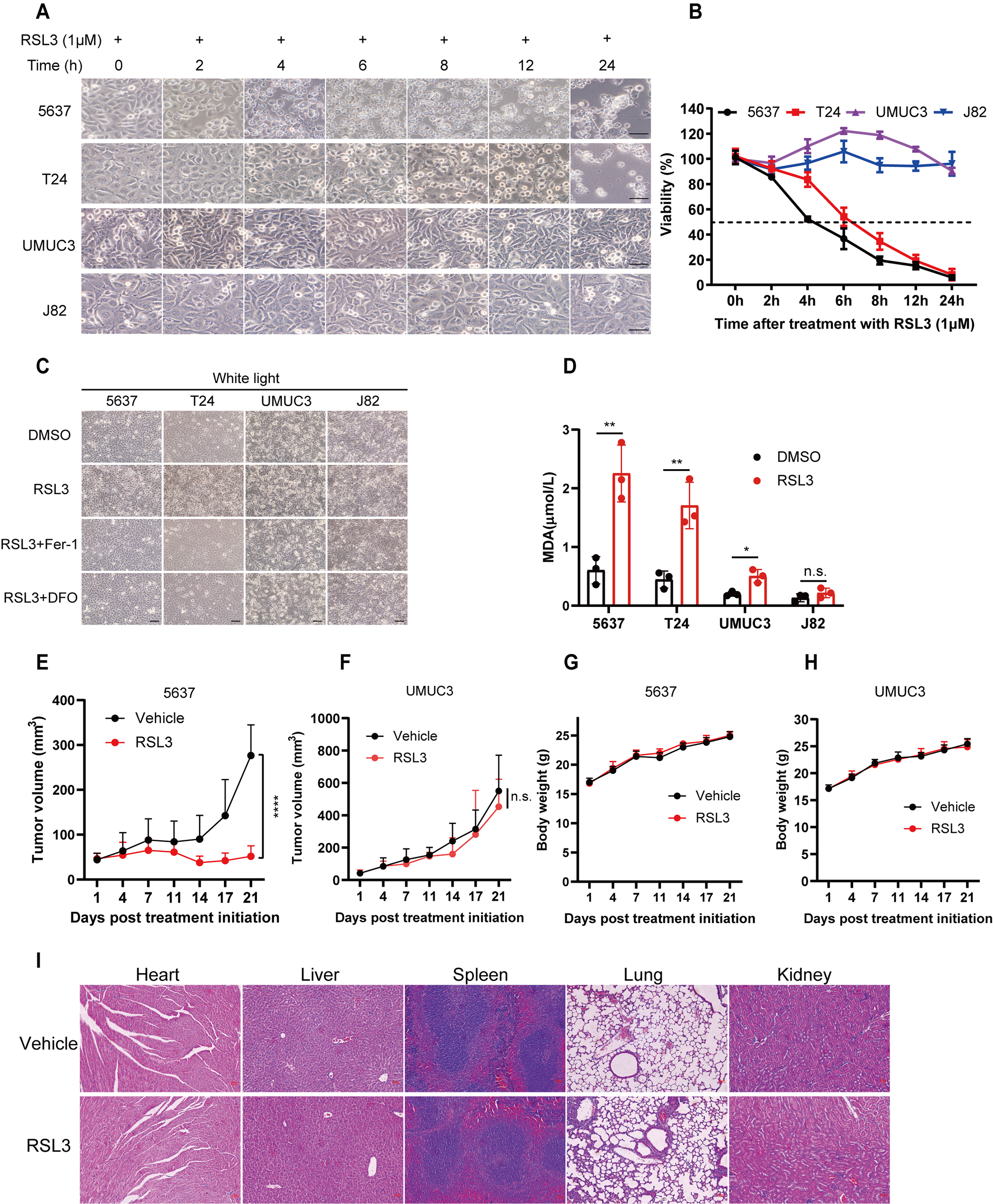

Supplement: Supplementary file 1 — Figure S1 [file 41419_2023_6333_MOESM1_ESM.tif]

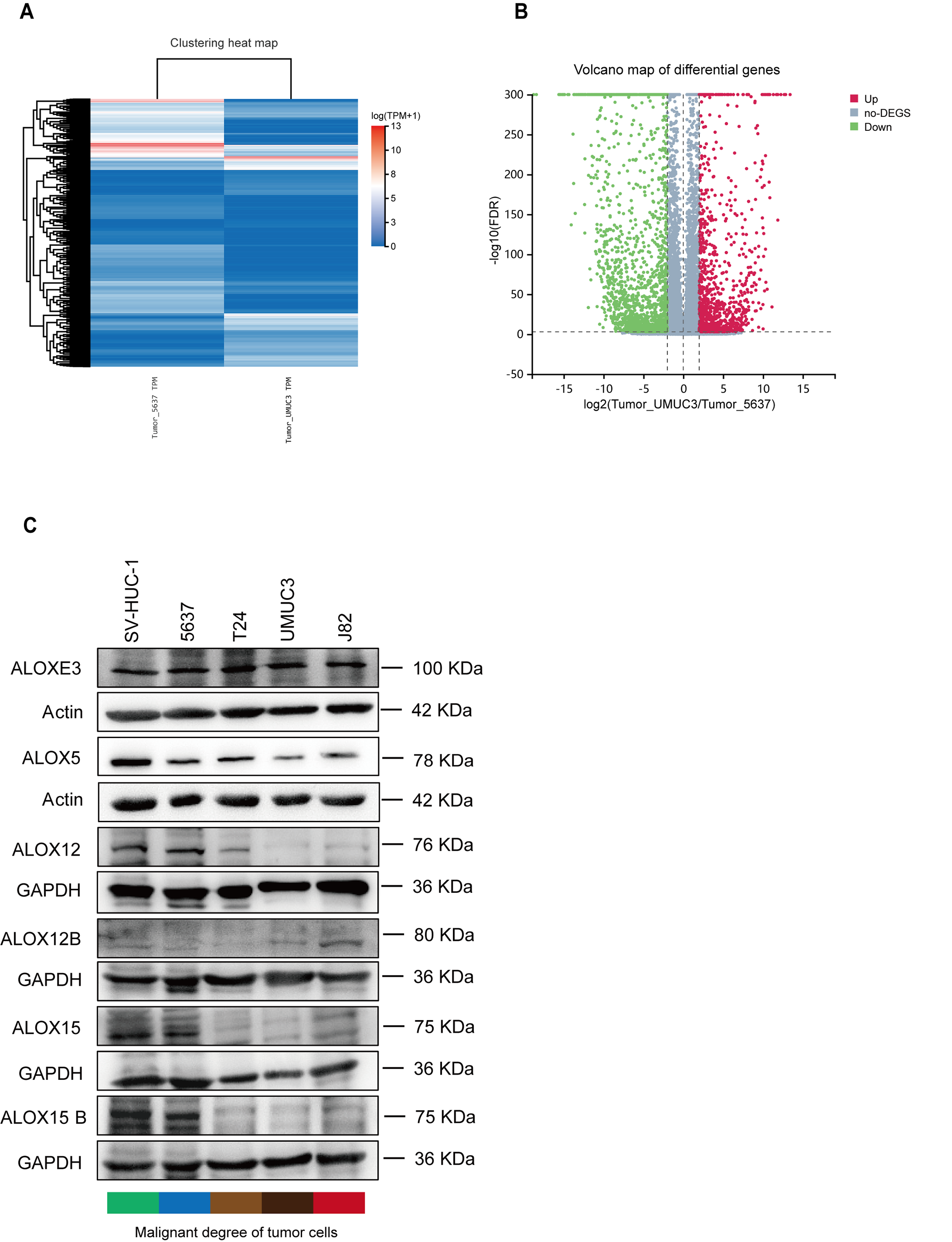

Supplement: Supplementary file 2 — Figure S2 [file 41419_2023_6333_MOESM2_ESM.tif]

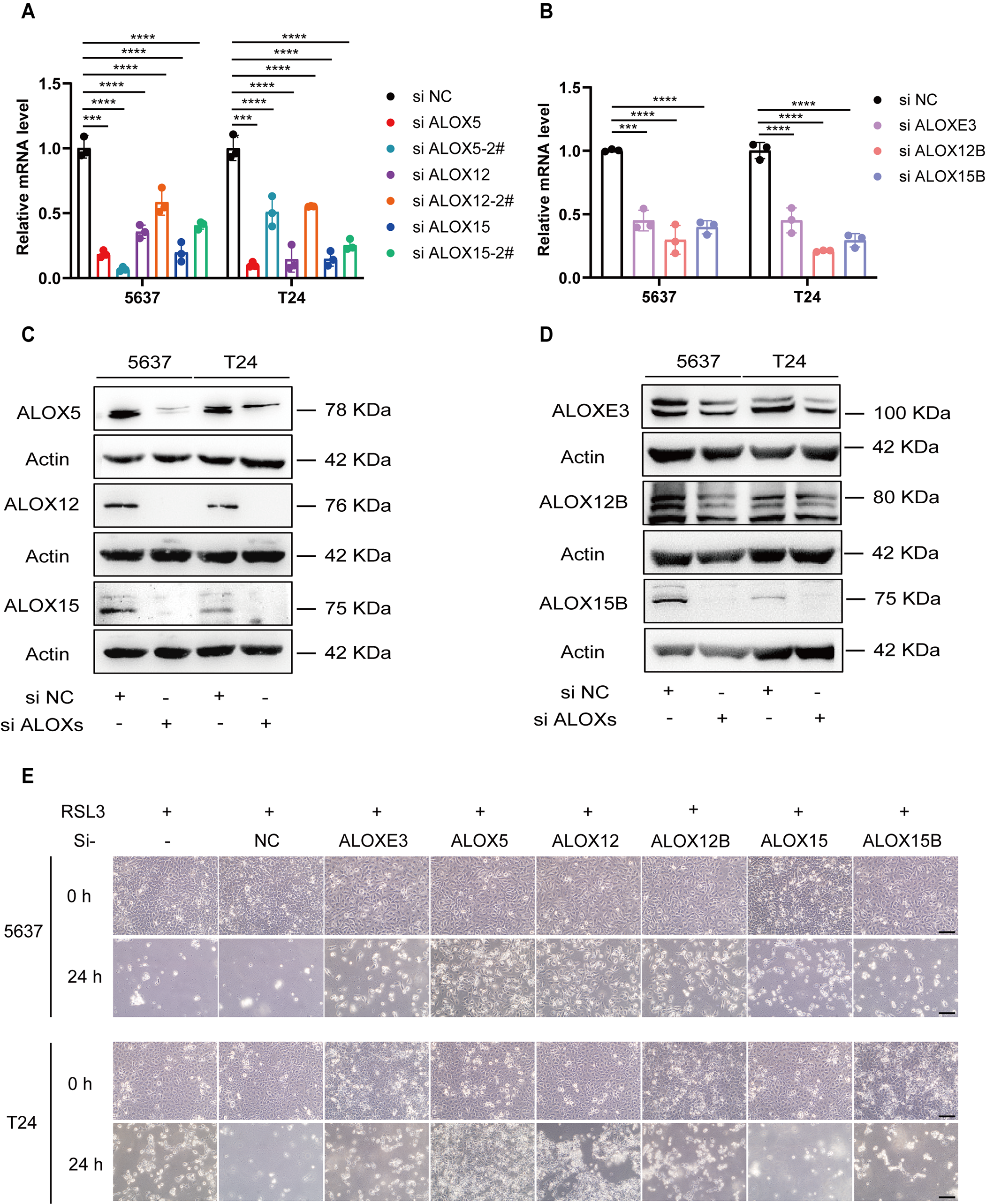

Supplement: Supplementary file 3 — Figure S3 [file 41419_2023_6333_MOESM3_ESM.tif]

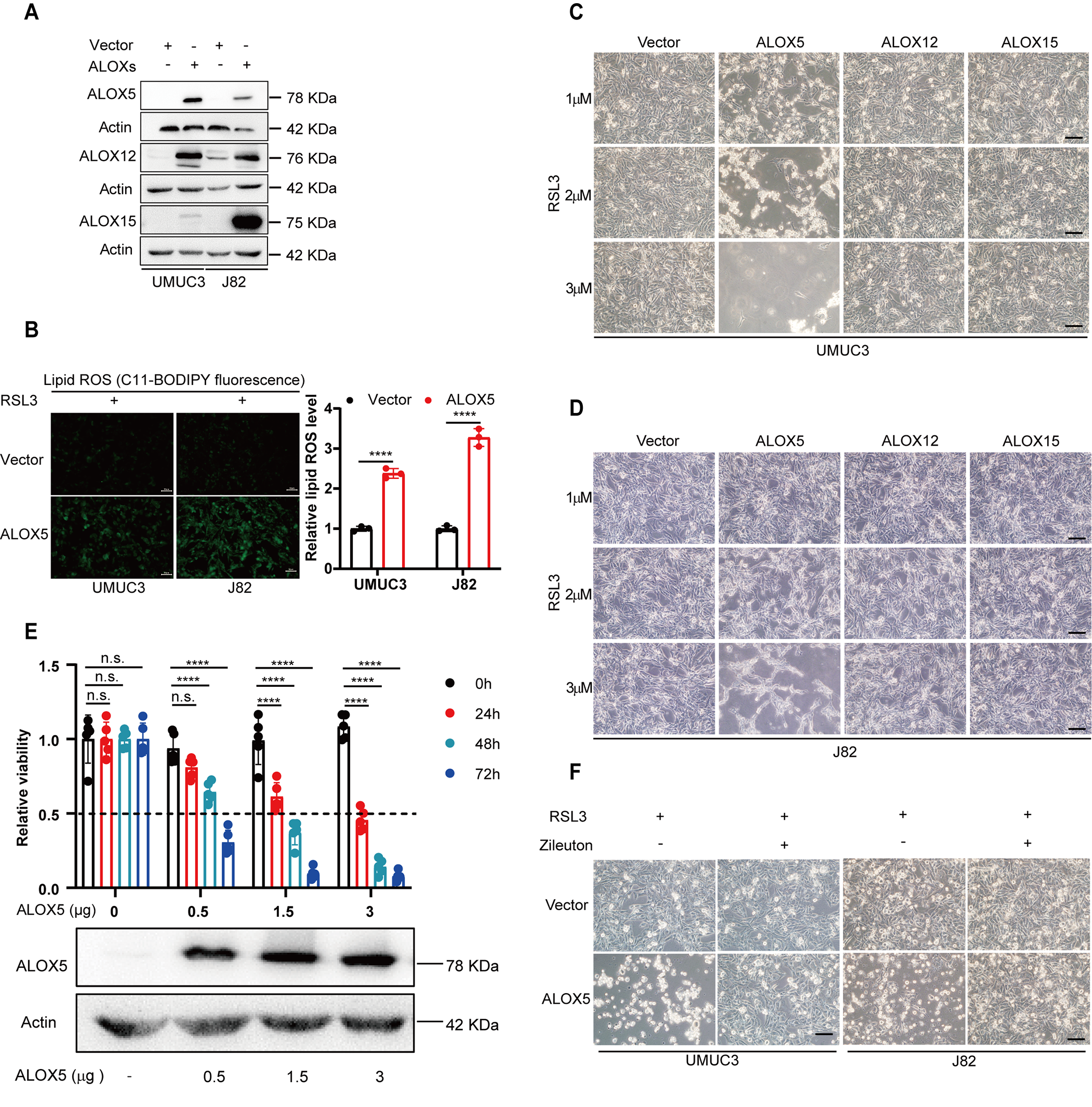

Supplement: Supplementary file 4 — Figure S4 [file 41419_2023_6333_MOESM4_ESM.tif]

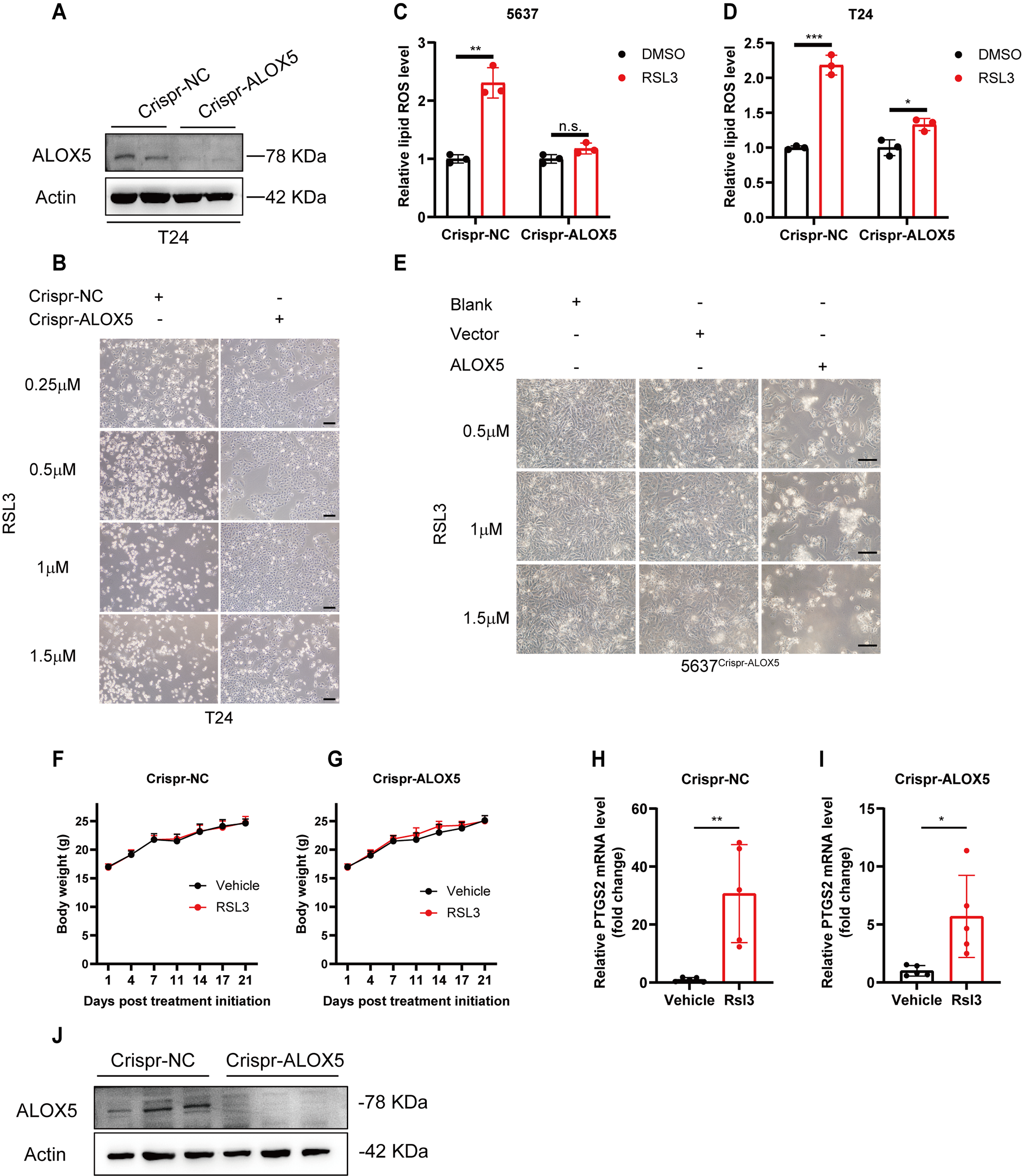

Supplement: Supplementary file 5 — Figure S5 [file 41419_2023_6333_MOESM5_ESM.tif]

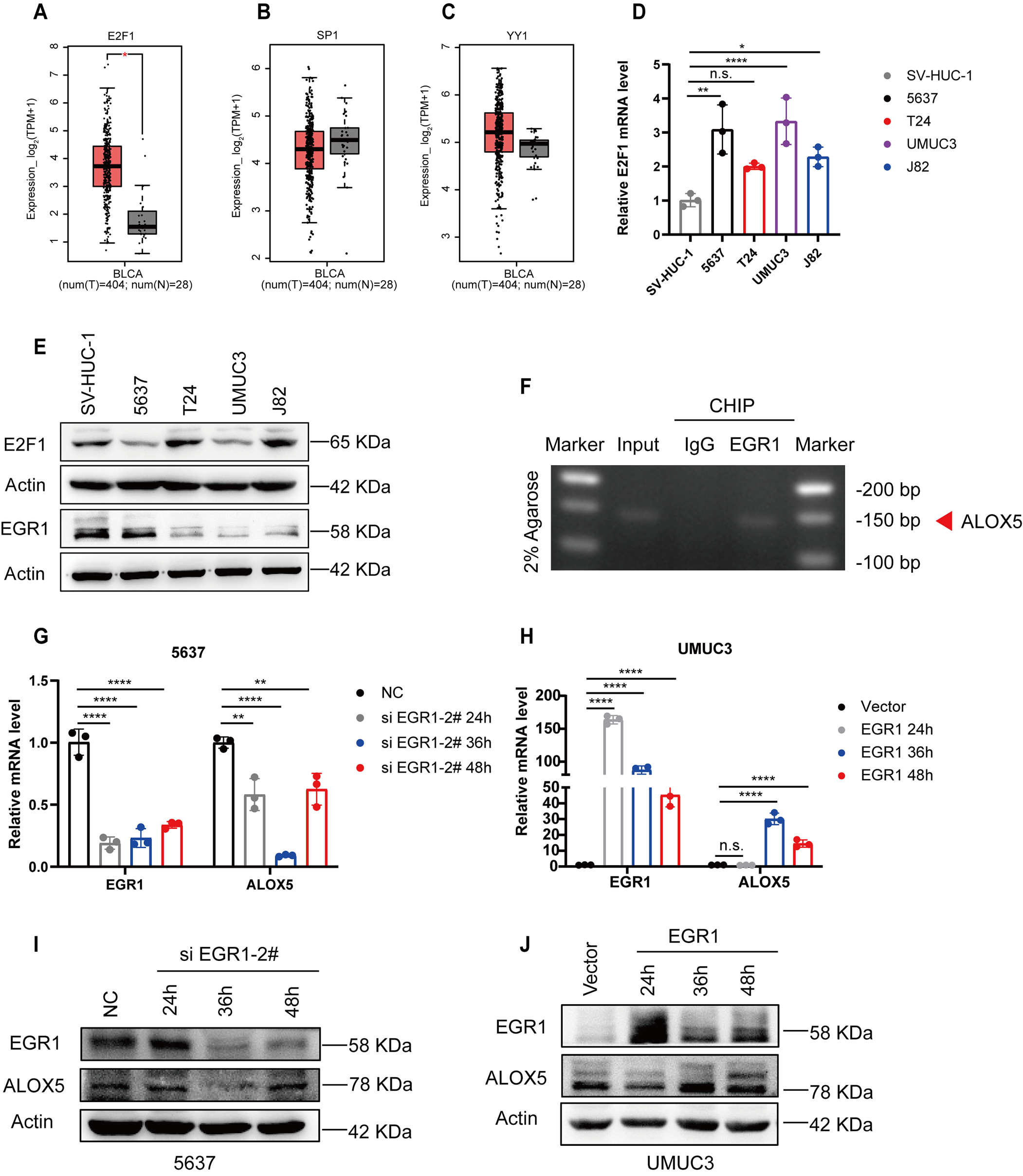

Supplement: Supplementary file 6 — Figure S6 [file 41419_2023_6333_MOESM6_ESM.tif]

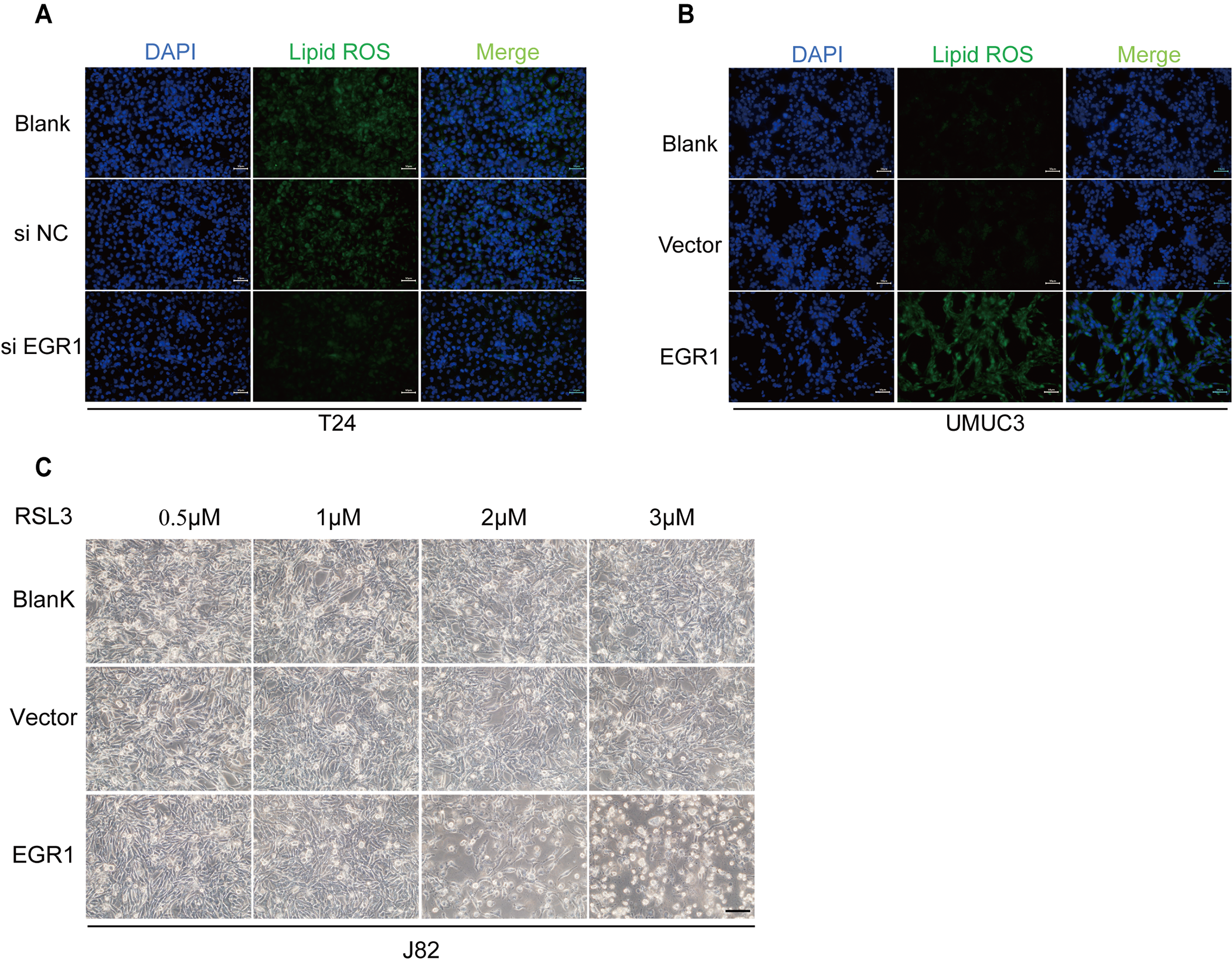

Supplement: Supplementary file 7 — Figure S7 [file 41419_2023_6333_MOESM7_ESM.tif]
