# Supplementary material for: ALOX5 deficiency contributes to bladder cancer progression by mediating ferroptosis escape
Source: Cell Death Dis. 2023 Dec 7;14(12):800. doi: 10.1038/s41419-023-06333-7 (PMC10703795; doi:10.1038/s41419-023-06333-7)
Supplement: Supplementary file 8 — Supplementary Figures legend [file 41419_2023_6333_MOESM8_ESM.docx]

**ALOX5 deficiency contributes to bladder cancer progression by mediating ferroptosis** **escape**

Tianyao Liu^1*^, Xinyan Xu^1*^, Jiazheng Li^2*^, Ming Bai^1*^, Wenjie Zhu^1^, Yanqing Liu^3^, Siyang Liu^1^, Zihan Zhao^1^, Tianhang Li^1^, Ning Jiang^4^, Yuhao Bai^4^, Qingyang Jin^1^, Yulin Zhang^1^, Yufeng Zheng^3^, Shengkai Zhou^3^, Shoubin Zhan^3^, Ying Sun^1^, Gaoli Liang^3^, Yang Luo^3^, Xi Chen^3†^, Hongqian Guo^1†^, Rong Yang^1, 2, 4†^

Affiliations: ^1^Nanjing Drum Tower Hospital, Affiliated Hospital of Medical School, Nanjing University, Nanjing, China.

^2^Department of Urology, Nanjing Drum Tower Hospital Clinical College of Nanjing University of Chinese Medicine, Nanjing, China

^3^Jiangsu Engineering Research Center for microRNA Biology and Biotechnology, State Key Laboratory of Pharmaceutical Biotechnology, School of Life Sciences, Nanjing University, Nanjing, China.

^4^Department of Urology, Nanjing Drum Tower Hospital Clinical College of Jiangsu University, Nanjing, China

**Supplementary materials: Figure S1-S7 legends**

**Fig. S1 High pathological stage BCa cells exist notably ferroptosis resistance. A** The time-dependant response of BCa to RSL3-induced ferroptosis. **B** Viability (%) of BCa cells treated with RSL3 (1 μM) at different time. Scale bars, 100 μm. **C** The withe light of BCa cells treated with RSL3 (1 μM) for 6 hours. Scale bars, 100 μm. **D** The MDA level of BCa cell lines after treatment with RSL3 (1 μM). **E-H** Tumor growth curve and body weight of xenograft tumor model after treatment with RSL3 (100 mg/kg) and vehicle. **I** HE staining of major organs. n.s., represent no significance, *p< 0.05, **p< 0.01, ****p< 0.0001. The data are represented as mean ± SD of three independent assays.

**Fig. S2** **Abnormal lipid metabolism may implicate in ferroptosis escape of BCa. A, B** RNA-seq and Volcanic plot analysis revealing differentially expressed genes (DEGs) in 5637 and UMUC3 cells. **C** Western blotting analysis the expression level of ALOXs in BCa cell lines.

**Fig. S3 RNAi-mediated ALOX5 deficiency promotes ferroptosis escape in BCa cells.** **A-D** qRT-PCR and western blotting analysis the expression level of ALOXs after RNAi-mediated depletion. **C, D** Representative images of RNAi-mediated ALOXs depletion BCa cells treated with RSL3 (1 μM) for 24 hours.

**Fig. S4** **Overexpression of ALOX5 sensitizes high-stage BCa cells to ferroptosis.** **A** Western blotting analysis the expression level of ALOXs after transfection with ALOXs. **B** The lipid ROS levels were measured using C11-BODIPY probe in UMUC3 and J82 cells. Scale bars, 50 μm. **C, D** Representative images of ALOX5 overexpression sensitizing ferroptosis in high-stage BCa cells. Scale bars, 100 μm. **E** UMUC3 cells transfected with different concentration overexpression plasmid of ALOX5 and then treated with RSL3 (2 μM) for different time. Viability was evaluated using CCK-8 kit. **F** Zileuton rescue ferroptosis caused by overexpressing ALOX5 in UMUC3 and J82 cells. UMUC3 treated with RSL3 (2 μM) for 48 hours. J82 treated with RSL3 (3 μM) for 48 hours. Zileuton, 40 μM. Scale bars, 100 μm. n.s., represent no significance, ****p< 0.0001. The data are represented as mean ± SD of three independent assays.

**Fig. S5 Knockout ALOX5 enhances BCa cells resistance to ferroptosis.** A) Western blotting analysis of the expression level of ALOX5 in T24 ^crispr-ALOX5^ cells. B) Representative images of T24 ^crispr-NC^ and T24 ^crispr-ALOX5^ cells treated with RSL3 (0.25, 0.5, 1, 1.5 μM) for 24 hours. Scale bars, 50 μm. C, D) Quantification analysis of the relative lipid ROS level in BCa cells after ALOX5 knockout using fluorescence intensity. E) Rescue experiment of re-expressing ALOX5 in knockout ALOX5 cells to reverse the ferroptosis resistance. Scale bars, 50 μm. F-I) Statistical analyses of body weight, PTGS2 expression levels. J) Western blotting confirmed the expression level of ALOX5 in xenograft tumor tissues. Scale bars, 50 μm. n.s., represent no significance, *p< 0.05, **p< 0.01, ***p< 0.001. The data are represented as mean ± SD.

**Fig. S6 EGR1 transcriptionally activates ALOX5 expression. A-C** The expression level of E2F1, SP1 and YY1 in TCGA database. **D** The expression level of E2F1 in BCa cell lines. **E** Western blot analysis of E2F1 and EGR1 protein levels in BCa cell lines. **F** ChIP results showing EGR1 binding on the ALOX5 promoter in 5637 cells. **G-H** Effects of knockdown or overexpression of EGR1 on ALOX5 expression. The most significant effect on ALOX5 was observed 36 hours after EGR1 transfection. n.s., represent no significance, *p< 0.05, **p< 0.01, ****p< 0.0001. The data are represented as mean ± SD of three independent assays.

**Fig. S7** **EGR1 mediates ferroptosis of BCa cells by transcriptional regulation of ALOX5.** **A, B** The lipid ROS level in T24 and UMUC3 was assessed by C11-BODIPY probe. Scale bars, 50 μm. **C** Representative images of J82 cells after transfection with EGR1 and then treated with RSL3. Scale bars, 50 μm.
