# Supplementary material for: ALOX5 deficiency contributes to bladder cancer progression by mediating ferroptosis escape
Source: Cell Death Dis. 2023 Dec 7;14(12):800. doi: 10.1038/s41419-023-06333-7 (PMC10703795; doi:10.1038/s41419-023-06333-7)
Supplement: Supplementary file 9 — Original Western blot data [file 41419_2023_6333_MOESM9_ESM.docx]

**ALOX5 deficiency contributes to bladder cancer progression by mediating ferroptosis** **escape**

Tianyao Liu^1*^, Xinyan Xu^1*^, Jiazheng Li^2*^, Ming Bai^1*^, Wenjie Zhu^1^, Yanqing Liu^3^, Siyang Liu^1^, Zihan Zhao^1^, Tianhang Li^1^, Ning Jiang^4^, Yuhao Bai^4^, Qingyang Jin^1^, Yulin Zhang^1^, Yufeng Zheng^3^, Shengkai Zhou^3^, Shoubin Zhan^3^, Ying Sun^1^, Gaoli Liang^3^, Yang Luo^3^, Xi Chen^3†^, Hongqian Guo^1†^, Rong Yang^1, 2, 4†^

Affiliations: ^1^Nanjing Drum Tower Hospital, Affiliated Hospital of Medical School, Nanjing University, Nanjing, China.

^2^Department of Urology, Nanjing Drum Tower Hospital Clinical College of Nanjing University of Chinese Medicine, Nanjing, China

^3^Jiangsu Engineering Research Center for microRNA Biology and Biotechnology, State Key Laboratory of Pharmaceutical Biotechnology, School of Life Sciences, Nanjing University, Nanjing, China.

^4^Department of Urology, Nanjing Drum Tower Hospital Clinical College of Jiangsu University, Nanjing, China

**Supplementary materials: Original Western blot images**


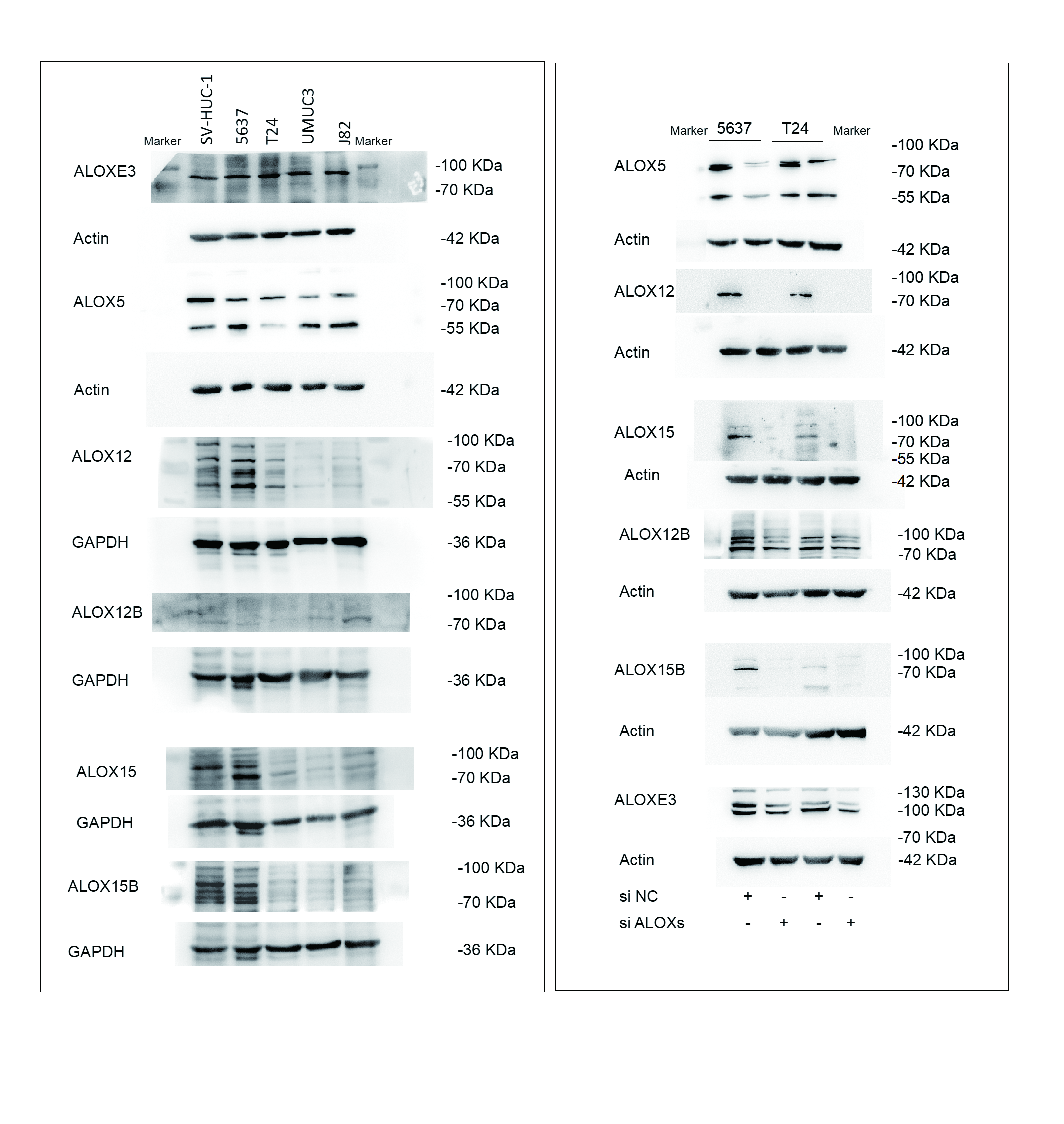


**
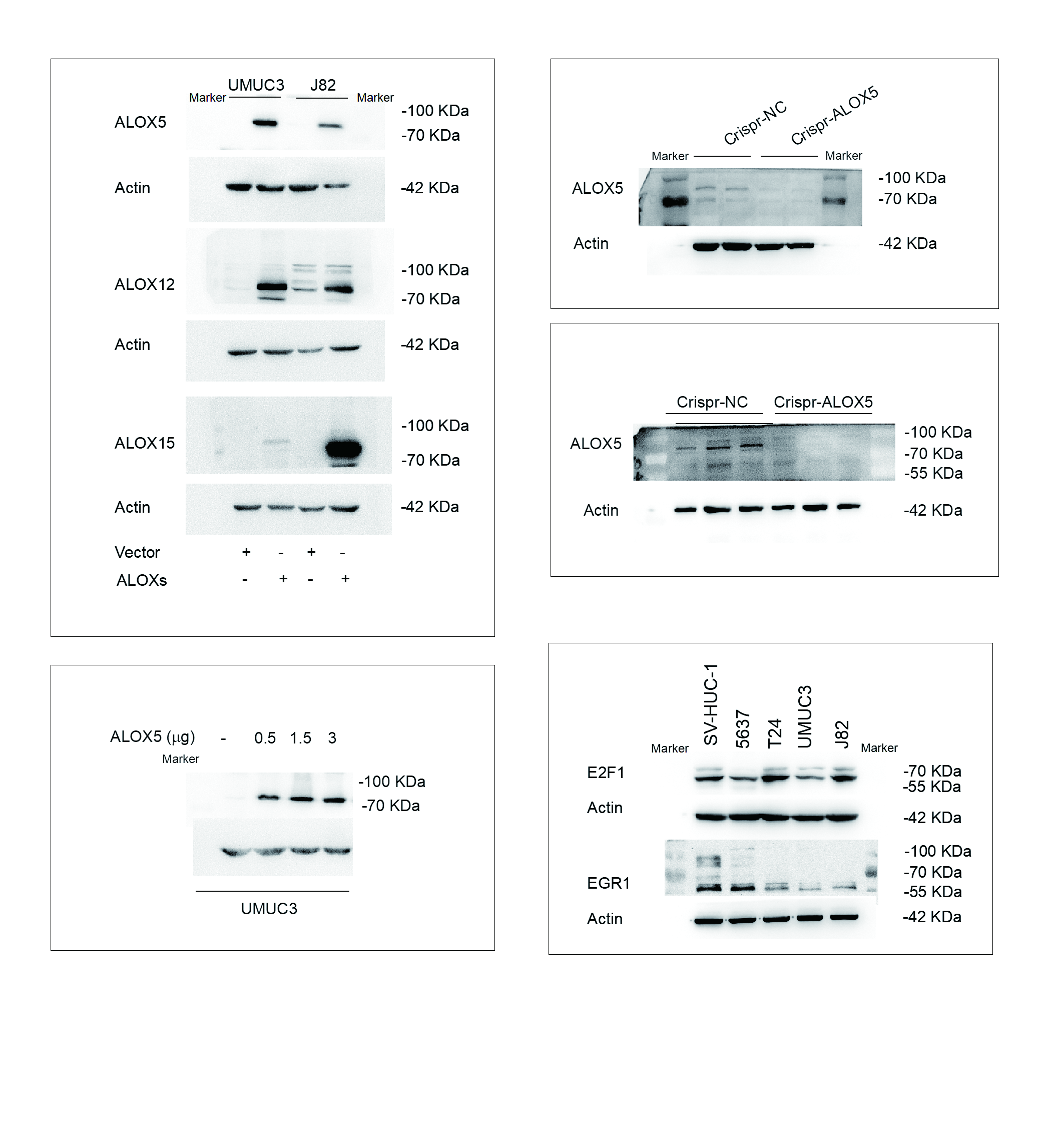
**

**
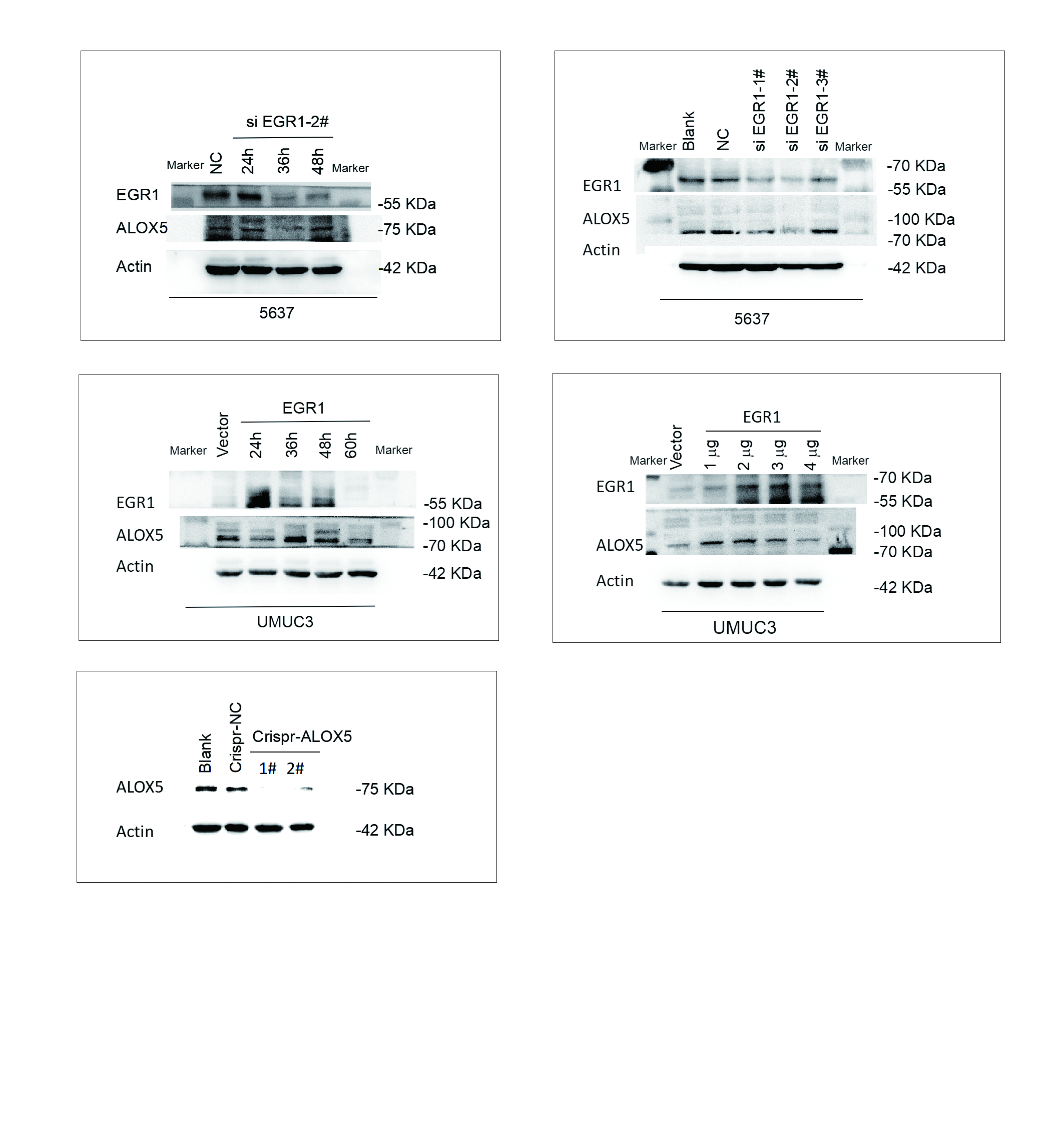
**
